# Delineating the molecular and phenotypic spectrum of the *SETD1B*-related syndrome

**DOI:** 10.1038/s41436-021-01246-2

**Published:** 2021-08-03

**Authors:** Marjolein J. A. Weerts, Kristina Lanko, Francisco J. Guzmán-Vega, Adam Jackson, Reshmi Ramakrishnan, Kelly J. Cardona-Londoño, Karla A. Peña-Guerra, Yolande van Bever, Barbara W. van Paassen, Anneke Kievit, Marjon van Slegtenhorst, Nicholas M. Allen, Caroline M. Kehoe, Hannah K. Robinson, Lewis Pang, Selina H. Banu, Mashaya Zaman, Stephanie Efthymiou, Henry Houlden, Irma Järvelä, Leena Lauronen, Tuomo Määttä, Isabelle Schrauwen, Suzanne M. Leal, Claudia A. L. Ruivenkamp, Daniela Q.C.M. Barge-Schaapveld, Cacha M. P. C. D. Peeters-Scholte, Hamid Galehdari, Neda Mazaheri, Sanjay M. Sisodiya, Victoria Harrison, Angela Sun, Jenny Thies, Luis Alberto Pedroza, Yana Lara-Taranchenko, Ivan K. Chinn, James R. Lupski, Alexandra Garza-Flores, Jeffery McGlothlin, Lin Yang, Shaoping Huang, Xiaodong Wang, Tamison Jewett, Gretchen Rosso, Xi Lin, Shehla Mohammed, J. Lawrence Merritt, Ghayda M. Mirzaa, Andrew E. Timms, Joshua Scheck, Mariet W. Elting, Abeltje M. Polstra, Lauren Schenck, Maura R. Z. Ruzhnikov, Annalisa Vetro, Martino Montomoli, Renzo Guerrini, Daniel C. Koboldt, Theresa Mihalic Mosher, Matthew T. Pastore, Kim L. McBride, Jing Peng, Zou Pan, Marjolein Willemsen, Susanne Koning, Peter D. Turnpenny, Bert B. A. de Vries, Christian Gilissen, Rolph Pfundt, Melissa Lees, Stephen R. Braddock, Kara C. Klemp, Fleur Vansenne, Marielle E. van Gijn, Catherine Quindipan, Matthew A. Deardorff, J. Austin Hamm, Abbey M. Putnam, Rebecca Baud, Laurence Walsh, Sally A. Lynch, Julia Baptista, Richard E. Person, Kristin G. Monaghan, Amy Crunk, Jennifer Keller-Ramey, Adi Reich, Houda Zghal Elloumi, Marielle Alders, Jennifer Kerkhof, Haley McConkey, Sadegheh Haghshenas, Genomics England Research Consortium, Reza Maroofian, Bekim Sadikovic, Siddharth Banka, Stefan T. Arold, Tahsin Stefan Barakat

**Affiliations:** 1 Department of Clinical Genetics, Erasmus MC University Medical Center, Rotterdam, The Netherlands; 2 King Abdullah University of Science and Technology (KAUST), Computational Bioscience Research Center (CBRC), Division of Biological and Environmental Sciences and Engineering (BESE), Thuwal, Saudi Arabia; 3 Manchester Centre for Genomic Medicine, St. Mary’s Hospital, Manchester University NHS Foundation Trust, Health Innovation Manchester, Manchester, UK; 4 Division of Evolution & Genomic Sciences, School of Biological Sciences, Faculty of Biology, Medicine and Health, University of Manchester, Manchester, UK; 5 Department of Paediatrics, National University of Ireland Galway, Galway, Ireland; 6 Exeter Genomics Laboratory, RILD Building, Royal Devon and Exeter NHS Foundation Trust, Exeter, UK; 7 Department of Pediatric Neurology, Dr. M.R. Khan Shishu (Children) Hospital and ICH, Mirpur, Dhaka, Bangladesh; 8 Department of Neuromuscular Disorders, Queen Square Institute of Neurology, University College London, London, UK; 9 Department of Medical Genetics, University of Helsinki, Helsinki, Finland; 10 Department of Clinical Neurophysiology, New Children’s Hospital, HUS Diagnostic Center, University of Helsinki and Helsinki University Hospital (HUH), Helsinki, Finland; 11 Disability Services, Joint Authority for Kainuu, Kajaani, Finland; 12 Center for Statistical Genetics, Sergievsky Center, Taub Institute for Alzheimer’s Disease and the Aging Brain, Department of Neurology, Columbia University Medical Center, New York, NY, USA; 13 Department of Clinical Genetics, Leiden University Medical Center, Leiden, The Netherlands; 14 Department of Neurology, Leiden University Medical Center, Leiden, The Netherlands; 15 Department of Genetics, Faculty of Science, Shahid Chamran University of Ahvaz, Ahvaz, Iran; 16 Department of Clinical and Experimental Epilepsy, UCL Queen Square Institute of Neurology, London, UK; 17 Chalfont Centre for Epilepsy, London, Bucks, UK; 18 Wessex Clinical Genetics Service, Princess Anne Hospital, Southampton, UK; 19 Department of Pediatrics, Division of Genetic Medicine, University of Washington School of Medicine, Seattle, Washington, USA; 20 Department of Pediatrics, Division of Genetic Medicine, Seattle Children’s Hospital, Seattle, WA, USA; 21 Department of Pediatrics, Vagelos College of Physicians and Surgeons, Columbia University Irving Medical Center, New York, New York, USA; 22 Universidad San Francisco de Quito, Colegio de ciencias de la salud-Hospital de los Valles, Quito, Ecuador; 23 Department of Pediatrics, Section of Immunology, Allergy, and Retrovirology, Baylor College of Medicine and Texas Children’s Hospital, Houston, TX, USA; 24 Center for Human Immunobiology of Texas Children’s Hospital/Department of Pediatrics, Baylor College of Medicine, Houston, TX, USA; 25 Baylor-Hopkins Center for Mendelian Genomics, Department of Molecular and Human Genetics, Baylor College of Medicine, Houston, TX, USA; 26 Human Genome Sequencing Center, Baylor College of Medicine, Houston, TX, USA; 27 Department of Pediatrics, Baylor College of Medicine, Texas Children’s Hospital, Houston, TX, USA; 28 Cook Children’s Genetics, Cook Children’s Physician Network, Cook Children’s Hospital, Fort Worth, TX, USA; 29 Cook Children’s Neurosciences, Cook Children’s Physician Network, Cook Children’s Hospital, Fort Worth, TX, USA; 30 Department of Pediatrics, The Second Affiliated Hospital of Xi ‘an Jiaotong University, Xi’an, China; 31 Cipher Gene Ltd, Beijing, China; 32 Department of Pediatrics, Section on Medical Genetics, Wake Forest School of Medicine, Winston-Salem, NC, USA; 33 Pediatrics Department, The First Affiliated Hospital of Fujian Medical University, Fuzhou, China; 34 Clinical Genetics, Guy’s and St Thomas NHS Foundation Trust, London, UK; 35 Center for Integrative Brain Research, Seattle Children’s Research Institute, Seattle, WA, USA; 36 Brotman Baty Institute for Precision Medicine, Seattle, WA, USA; 37 Center for Developmental Biology and Regenerative Medicine, Seattle Children’s Research Institute, Seattle, WA, USA; 38 Department of Clinical Genetics, VU University Medical Center, Amsterdam, The Netherlands; 39 Department of Pediatrics, Division of Medical Genetics, Stanford Medicine, Stanford, CA, USA; 40 Department of Neurology and Neurological Sciences, Stanford Medicine, Stanford, CA, USA; 41 Pediatric Neurology, Neurogenetics and Neurobiology Unit and Laboratories, Meyer Children’s Hospital-University of Florence, Florence, Italy; 42 Nationwide Children’s Hospital, Columbus, OH, USA; 43 Xiangya Hospital of Central South University, Changsha, China; 44 Department of Human Genetics, Radboud university medical center, Nijmegen, The Netherlands; 45 Department of Clinical Genetics, Maastricht University Medical Center, Maastricht, The Netherlands; 46 Clinical Genetics, Royal Devon & Exeter NHS Foundation Trust, Exeter, UK; 47 NE Thames Regional Genetics Service, Great Ormond Street Hospital for Children NHS Foundation Trust, London, UK; 48 Division of Medical Genetics, Department of Pediatrics, SSM Health Cardinal Glennon Children’s Hospital, Saint Louis University School of Medicine, Saint Louis, MO, USA; 49 Department of Clinical Genetics, University Medical Center Groningen, Groningen, The Netherlands; 50 Center for Personalized Medicine, Department of Pathology and Laboratory Medicine, Children’s Hospital Los Angeles, Los Angeles, CA, USA; 51 Departments of Pathology and Pediatrics, Keck School of Medicine of the, University of Southern California, Los Angeles, CA, USA; 52 East Tennessee Children’s Hospital Genetics Center, Knoxville, TN, USA; 53 Department of Medical and Molecular Genetics, Indiana University School of Medicine, Indianapolis, IN, USA; 54 Department of Neurology, Indiana University School of Medicine and Riley Hospital for Children, Indianapolis, IN, USA; 55 Department of Clinical Genetics, Children’s Health Ireland at Temple St. Children’s Hospital and Our Lady’s Children’s Hospital, Crumlin, Dublin, Ireland; 56 Institute of Biomedical and Clinical Science, University of Exeter Medical School, Exeter, UK; 57 GeneDx, Gaithersburg, MD, USA; 58 Amsterdam UMC, Department of Clinical Genetics, Amsterdam Reproduction & Development Research Institute, University of Amsterdam, Amsterdam, The Netherlands; 59 Molecular Genetics Laboratory, Molecular Diagnostics Division, London Health Sciences Centre, London, ON, Canada; 60 Department of Pathology and Laboratory Medicine, Western University, London, ON, Canada; 61 Centre de Biologie Structurale, CNRS, INSERM, Université de Montpellier, Montpellier, France

## Abstract

**Purpose:**

Pathogenic variants in *SETD1B* have been associated with a syndromic neurodevelopmental disorder including intellectual disability, language delay, and seizures. To date, clinical features have been described for 11 patients with (likely) pathogenic *SETD1B* sequence variants. This study aims to further delineate the spectrum of the SETD1B-related syndrome based on characterizing an expanded patient cohort.

**Methods:**

We perform an in-depth clinical characterization of a cohort of 36 unpublished individuals with *SETD1B* sequence variants, describing their molecular and phenotypic spectrum. Selected variants were functionally tested using in vitro and genome-wide methylation assays.

**Results:**

Our data present evidence for a loss-of-function mechanism of *SETD1B* variants, resulting in a core clinical phenotype of global developmental delay, language delay including regression, intellectual disability, autism and other behavioral issues, and variable epilepsy phenotypes. Developmental delay appeared to precede seizure onset, suggesting SETD1B dysfunction impacts physiological neurodevelopment even in the absence of epileptic activity. Males are significantly overrepresented and more severely affected, and we speculate that sex-linked traits could affect susceptibility to penetrance and the clinical spectrum of *SETD1B* variants.

**Conclusion:**

Insights from this extensive cohort will facilitate the counseling regarding the molecular and phenotypic landscape of newly diagnosed patients with the SETD1B-related syndrome.

## Introduction

**Fig. 1:**
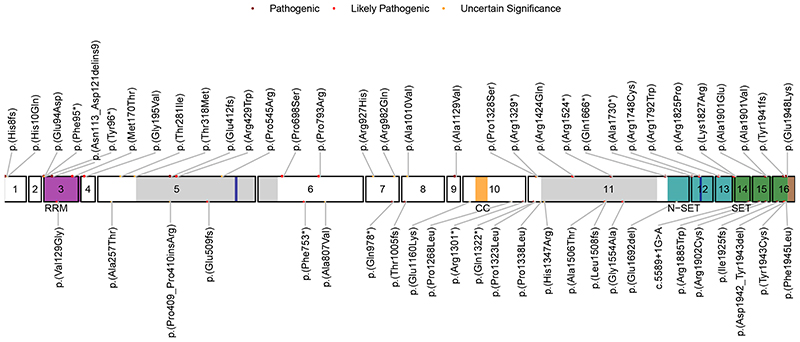
Schematic representation of *SETD1B* variants in this study cohort (major circles, top labels) and in literature (minor circles, bottom labels). The RRM (residues 94–182), coiled-coil (CC) (residues 1173–1204), N-SET (residues 1668–1821), SET (residues 1822–1948) and post-SET (residues 1949–1966) domains in respectively magenta, orange, cyan, green and brown, the largely disordered regions (residues 320–682 and residues 1338–1640) in light gray, and the LSD (exon 5, residues 577–583) and WIN (exon 12, residues 1745–1750, within N-SET) motifs both in blue.

*SETD1B* encodes a lysine-specific histone methyltransferase that methylates histone H3 at position lysine-4 (H3K4me1, H3K4me2, H3K4me3) as part of a multisubunit complex known as COMPASS [1, 2]. The SETD1B protein consists of 1,966 amino acids and has several (presumed) functional domains (Fig. 1). The N-terminus contains an RNA recognition motif (RRM), whereas the middle region is characterized by two long disordered regions that differ from other homologs [3, 4], a conserved lysine–serine–aspartic acid (LSD) motif [5] and a coiled-coil structure. At the C-terminus, SETD1B harbors a catalytic SET domain crucial for histone methyltransferase activity, bordered proximally by the N-SET domain including a conserved WDR5-interacting (WIN) motif [6], and distally by the post-SET domain. H3K4me3 is enriched at promoter and transcription start sites whereas H3K4me1 and H3K4me2 are enriched at enhancer sites, therefore being associated with active gene transcription and euchromatin [7]. Indeed epigenetic changes have been observed in both animal models and patient material [8–10] at promoters and intergenic regions, confirming that SETD1B epigenetically controls gene expression and chromatin state. In addition, *SETD1B* is constrained for both missense and loss-of-function variants [11].

Consistent with this, pathogenic variants in *SETD1B* have been associated with a syndromic intellectual developmental disorder including seizures and language delay (IDDSELD, OMIM 619000). To date, clinical features have been described for 11 affected individuals with (likely) pathogenic *SETD1B* sequence variants [8, 12–15]. Individuals with microdeletions encompassing *SETD1B* have also been described [8, 16–19]; however, most of these deletions encompass additional genes making phenotypic comparisons challenging. In this study, we further delineate the clinical phenotype associated with *SETD1B* sequence variants, by describing 36 additional individuals. Comparing these new cases to the published ones provides a comprehensive molecular and clinical characterization of the *SETD1B*-related syndrome. In addition, using protein modeling, in vitro assays, and genome-wide methylation signatures we investigate the effects of selected variants. Together, this expands the molecular and phenotypic landscape associated with *SETD1B* variants.

## Materials and Methods

### Cohort inclusion

After identification of three individuals with *SETD1B* variants at Erasmus MC Clinical Genetics, additional cases were identified using GeneMatcher [20] and the Dutch Datasharing Initiative [21] and via our network of collaborators. Individuals were included based on *SETD1B* variants detected in research or routine clinical diagnostics. Affected individuals were investigated by their referring physicians.

### Next-generation sequencing of affected individuals

Full details are provided in the Supplementary Methods and Supplementary Fig. S1.

### Variant classification

*SETD1B* variants were initially classified as variants of uncertain significance (VUS), likely pathogenic, or pathogenic at the performing laboratory or local referring sites. Literature and public database search identified 30 individuals with *SETD1B* sequence variants (Supplementary Table S1). Reclassification of *SETD1B* sequence variants was performed according to American College of Medical Genetics and Genomics/Association for Molecular Pathology (ACMG/AMP) Standards and Guidelines [22] (Supplementary Table S1), using reference sequence NM_001353345. For retrieval of population allele frequencies and in silico predictions Alamut® Visual 2.15 (Feb 2020) was used.

### Facial gestalt and severity scoring analysis

To generate a composite facial gestalt, Face2Gene (FDNA Inc., Boston, MA, USA) research application was used (default settings). Details of severity scoring are described in Supplementary Methods.

### Structural protein modeling

Sequences were retrieved from Uniprot, SWISS-MODEL [23] to produce homology models; RaptorX [3] for predicting secondary structure and disorder; ConSurf [24] for conservation analysis; and eukaryotic linear motif (ELM [25]) for short linear protein motif assessment. Models were manually inspected, and variants evaluated, using Pymol (pymol.org).

### Experimental procedures

For in vitro experiments flag-tagged wild type (kindly provided by David Skalnik, Indiana University [5]) and variant SETD1B protein and HA-tagged ASH2 were overexpressed in HEK293 cells. Protein expression, isolation, western blotting, and immunocytochemistry were performed following standard procedures [26–28]. Genome-wide methylation profiles were obtained as described [8]. Details on experimental procedures and statistical analysis are provided in Supplementary Methods.

## Results

### Molecular spectrum of *SETD1B* sequence variants

A total of 36 individuals with either heterozygous (*n*=32, *n*=28 confirmed de novo, n=1 inherited from affected parent, n=1 inherited from unaffected parent), compound heterozygous (*n*=2, biallelic inheritance from unaffected parents) or homozygous (*n*=2, siblings, biallelic inheritance from unaffected parents) *SETD1B* sequence variants were included in this cohort. Thirty-three variants were detected, of which 2 were recurrent. This includes 8 truncating (*n*=6 nonsense, *n*=2 frameshift), 1 extension, 1 in-frame inversion, and 23 missense variants (Fig. 1). Fourteen variants were classified as pathogenic, ten as likely pathogenic, and nine as uncertain significance. For individuals with VUS, no alternative candidate disease-causing variant was identified. In literature, 26 additional (4 recurrent) *SETD1B* variants have been reported including 7 truncating, 1 splicing, 1 extension, 3 in-frame insertions or deletions, and 14 missense variants (Fig. 1). Variants are distributed along the protein (Fig. 1), with the majority of (likely) pathogenic missense variants located within the SET domain region.

### Clinical spectrum

**Fig. 2:**
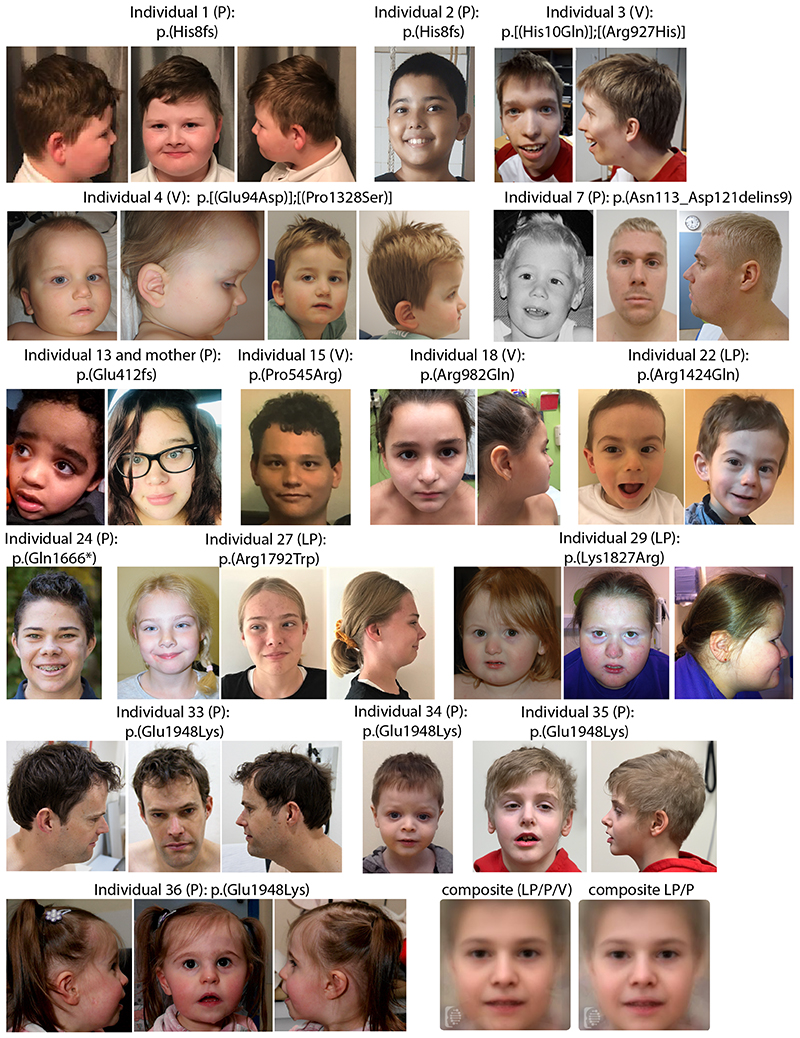
Facial images of affected individuals. Photographs of 16 individuals (plus one affected mother) with indicated *SETD1B* variants. Dysmorphic features included, among others, a slightly elongated face, high anterior hairline, thick arched or straight eyebrows, deep-set eyes, a prominent nose, and thin upper lips. Lower right corner shows facial composite images for all individuals, or only those with a likely pathogenic or pathogenic variant (note: individual 13 and mother were not included in the composite, given the image angle and glasses hindering Face2Gene program analysis). LP likely pathogenic variant, P pathogenic variant, V variant of uncertain significance.

The cohort consists of 24 males and 12 females, whose age at last evaluation ranged from 1 to 44 years (median 9 years, interquartile range [IQR] 6–15 years). Table 1 gives an overview of the core clinical phenotype, and Fig. 2 displays the facial appearance of individuals for whom photographs were available. The phenotype of individuals with VUS (either biallelic or heterozygous) matched that of the overall cohort (Table 2). More details can be found in Supplementary Case Reports and Supplementary Fig. 2–4.

### Development and neurological findings

Most individuals were born after an uneventful pregnancy at full term, with an unremarkable neonatal period and anthropomorphic measurements in the normal range. Seven individuals (7/31, 23%) had postnatal hypoglycemia. Virtually all individuals (34/36, 94%) showed global developmental delay in early infancy. Notably, individuals 14 and 16 without documented developmental delay are the youngest individuals (respectively 2 and 1 years old). Motor development was delayed in 32 individuals (32/ 36, 89%), with independent ambulation acquired between 1.0 and 4.5 years of age (median 1.6, IQR 1.3–2.5, one individual is nonambulatory). Motor performance remained an issue, with clumsiness, coordination difficulties, and poor fine motor movements reported. Hypotonia was documented for 16 individuals (16/35, 46%), often manifesting in neonatal or childhood period. Language development was delayed in the majority of individuals (33/36, 92%), with first words acquired between 0.5 and 3.0 years (median 2.0, IQR 1.1–2.1). Five individuals were nonverbal at time of data collection (15%, respectively 2.5, 3, 3.5, 11, and 19 years old), and at least five additional individuals (15%) speak far fewer words than appropriate for their age. Regression of previously acquired skills was reported in nine individuals, especially with regard to language, without an obvious link to epileptic activity. At the last investigation, intellectual disability was present in 28 individuals (28/32, 88%), ranging from mild (*n* = 9), to moderate (*n* = 8) and severe (*n* = 4) (not specified *n* = 7). Formal IQ testing was performed in 11 individuals with an average score of 60 (IQR 48–67) (mild). Autistic features were observed in 24 individuals (24/36, 67%); other behavioral issues included hyperactivity (13/ 34, 38%), sleep disturbance (10/32, 31%), anxiety (11/35, 31%), anger or aggressive behavior (11/35, 31%, including self-mutilation for individuals 4 and 10), and obsessive compulsive behavior (individual 7, 26, 30). Epilepsy developed in 28 individuals (28/36, 78%) with a median age of seizure onset of 3 years (IQR 1.0–5.3). Eight individuals remained seizure-free up to an age of 16 years (range 2–16, median 6.0, IQR 4.5–7.3 years). At their onset, the majority of classifiable seizures were generalized (*n* = 19) and minority focal (*n* = 5), and included motor (*n* = 9) or nonmotor (*n* = 13) involvement, with variable development into seizure types over time (Table 1). Seizure frequency varied (sporadic to very frequent) and was at least daily in the majority of patients. Fever-sensitive seizures were reported in three individuals. Whereas seizures were (partially) controlled using various antiepileptic drugs in eighteen individuals, seizures responded poorly or remained intractable in seven individuals. Brain MRI (Supplementary Fig. S2) was performed in 33 individuals and was often unremarkable (23/31, 74%). Abnormal MRI findings included nonspecific minor subcortical white matter hyperintensities (individual 1); cystic encephalomalacia with ventriculomegaly (individual 4); reduced white matter volume and thin corpus callosum (individual 10); bilateral abnormal signals at frontal, temporal, and occipital lobes (individual 16); extensive irregular gyral pattern with reduced sulcation (individual 19); slightly delayed myelination and small heterotopic gray matter (individual 21); periventricular leukomalacia (individual 30, possible due to an underlying hypoplastic left heart disease); and mild diffuse cerebral volume loss with ex vacuo enlargement of lateral and third ventricles (individual 32).

### Additional findings

Ophthalmological findings included strabismus (*n* = 5), amblyopia (*n* = 2), myopia (*n* = 2), astigmatism (*n* = 3), and cortical vision impairment (*n* = 1). Eight individuals showed gastrointestinal symptoms, including reflux, constipation, and feeding problems. Ten individuals had dermatological symptoms (eczema, rough or dry skin, café au lait spots, hypo- or hyperpigmentation). A number of individuals displayed skeletal abnormalities (scoliosis [*n* = 5], kyphosis [*n* = 2], joint hypermobility [*n* = 4]). (Recurrent) respiratory and urinary tract infections were reported in six individuals. No malignancies were identified. (Truncal) overweight or obesity was present in 17 individuals (Supplementary Table S2).

### Facial appearance

Facial appearance varied from no discernible (5 individuals) to mild dysmorphic features (31 individuals, 86%) (Fig. 2). Dysmorphisms included prominent rounded nasal tip/bulbous nose (*n* = 15), high anterior hairline (*n* = 11), (uplifted) large earlobes (*n* = 10), overfolded superior helices (*n* = 6), low-set ears (*n* = 5), thin upper lip (*n* = 9), pointed/prominent chin (*n* = 6), deep-set eyes (*n* = 5), synophrys (*n* = 4), full cheeks (*n* = 4), elongated/narrow face (*n* = 5) and/or bitemporal narrowing (*n* = 4), and frontal bossing (*n* = 4). Also, tapering fingers (*n* = 5), brachydactyly (*n* = 3), small hands (*n* = 5), and nail hypoplasia (*n* = 4) were reported (Supplementary Fig. S3).

### Structural modelling of variants

**Fig. 3:**
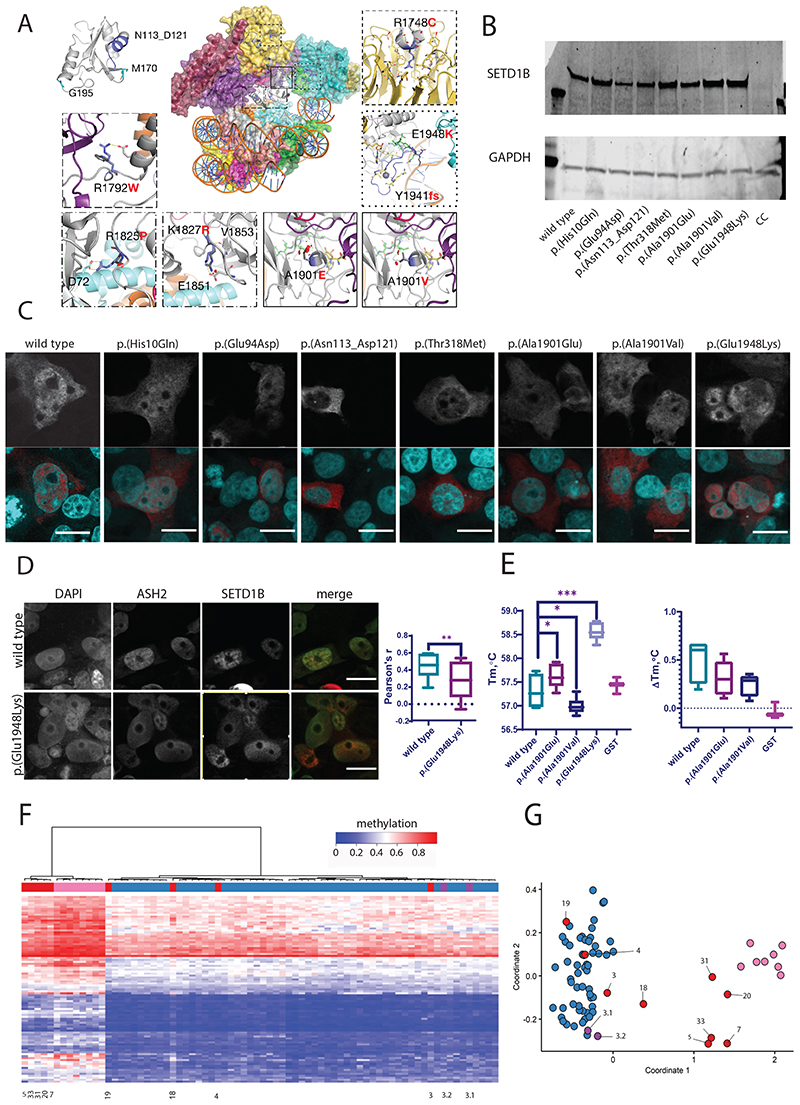
Structural and functional evaluation of SETD1B variants. (**a**) Homology models of SETD1B domains: RRM domain (top left), based on the crystal structure of the RRM of human SETD1A (PDB ID 3S8S, identity = 66%, QMEAN = 0.25). The segment of Asn113 to Asp121 is colored in blue. This region is known to support different protein–protein interactions in other RRM proteins. Met170 and Gly195 are shown as blue sticks. Homology model of the N-SET and catalytic SET domains of SETD1B (gray cartoon), based on the EM structure of the yeast COMPASS in a complex with a ubiquitinated nucleosome (PDB ID 6ven, identity= 40.21%, QMEAN = −5.10) (center superimposed to the template PDB, and zoom-in panels). The region containing Arg1748 was observed more accurately in the X-ray structure of the WDR5:SETD1B Win motif peptide binary complex (PDB ID 4es0 [6], top right): Arg1748 (blue sticks) is inserted into the pocket of WDR5 (yellow) and interacts with the backbone oxygen atoms of Ser91, Phe133 and Cys261 (hydrogen bonds shown as yellow dashed lines). Arg1792 (blue sticks) and the substitution by Trp (dark gray sticks) interacting with surrounding residues in the adjacent alpha helix (e.g., Glu1796, gray sticks) or with the SWD1 subunit (RBBP5 in humans, shown in violet). The insets with Arg1825 and Arg1827 show the proximity of these residues (dark blue sticks) to histone H2A (light blue cartoon). The SET domain containing Ala1901Val, Ala1901Glu, Tyr1941fs and Glu1948Lys was modeled more accurately based on the crystal structure of the yeast COMPASS catalytic module (PDB ID 6chg [40], identity=62%, QMEAN = −1.78). Ala1901 and Glu1948 are presented as blue sticks in the center figure and right insets. The Ala1901Val and Ala1901Glu substitutions (dark gray sticks) could compromise the stability of the adjacent SAM (olive sticks) binding site and the interaction with the SWD1 subunit (RBBP5 in humans, violet cartoon), which in turn contacts ubiquitin (red cartoon). Tyr1941fs alters a segment of SET and Post-SET regions involved in catalysis and cofactor binding (blue cartoon in center figure and right inset): SAM (olive sticks) and histone H3 (green sticks) binding pocket, the key Tyr1943 residue (yellow sticks), three Cys and one Arg (yellow sticks) coordinating a zinc atom (shown as a sphere). The Glu1948Lys substitution (blue/dark gray sticks in center figure and right inset) could disturb potential interactions between the flexible loops and the adjacent subunit (Bre2, homologous to human ASH2, is shown in teal cartoon). (**b**) Overexpression of wild-type and variant SETD1B protein in HEK293 cells 48 hours post-transfection assessed by western blot. CC cell control, lysate of mock transfected HEK293 cells (one-way analysis of variance [ANOVA] *p* = 0.09). (**c**) Nuclear localization of SETD1B variants in HEK293 cells. Upper panel—SETD1B detected by anti-Flag antibody; lower panel—overlay of nuclear staining (DAPI, cyan) and SETD1B (red); scale bar 20 μm. Images representative of 2 independent experiments are shown. (**d**) Colocalization of SETD1B and ASH2 in HEK293 cells. Left to right: nuclear staining (DAPI), ASH2 (anti-HA tag), SETD1B (anti-Flag tag), merge of ASH2 (green) and SETD1B (red); scale bar 20μm. Pearson’s r value (range: −1, negative correlation, 1, max correlation) calculated with coloc2 plugin (ImageJ), Z-stacks of min. 12 nuclei were used for the analysis. *t*-test **p = 0.005. (**e**) Thermal shift analysis of the SET domain. Left: *T_m_* of GST-SETD1B proteins and GST control. Right: change in *T_m_* of the proteins in presence of SAM substrate. Two independent protein preparations were used for the assay performed in triplicates. One-way ANOVA multiple comparison test *p < 0.05, ***p < 0.0001. (**f**,**g**) Analysis of methylation profiles. (**f**) Hierarchical clustering (rows represent methylation probes, columns–samples). (**g**) MDS plot (control samples in blue, proband samples in red, SETD1B cases from the database in pink). Sample numbers correspond to case numbers: individual 3 p.([His10Gln];[Arg927His]) (3.1 and 3.2 are the parents of individual 3), individual 4 p.([Glu94Asp];[Pro1328Ser]), individual 5 p.(Phe95*), individual 7 p.(Asn113_Asp121delins9), individual 18 p.(Arg982Gln), individual 19 p.(Ala1010Val), individual 20 p.(Ala1129Val), individual 31 p.(Ala1901Glu), and individual 33 p.(Glu1948Lys).

The eight truncating variants (p.[His8fs], p.[Phe95*], p.[Tyr96*], p.[Glu412fs], p.[Arg1329*], p.[Arg1524*], p.[Gln1666*], p.[Ala1730*]) are likely to be targeted for nonsense-mediated decay, but if not would result in removal of the SET region eliminating catalytic activity. Variants p.(His10Gln) and p.(Glu94Asp) are located in a disordered region preceding the RRM (Figs. 1 and 3a) and could affect the specificity of the potential interactions mediated by RRM’s N-terminus [28]. The nucleotide inversion leading to p.(Asn113_Asp121delins9) and substitution p.(Met170Thr) are located in the canonical β_1_α_1_β_2_β_3_α_2_β_4_ RRM region, whereas p.(Gly195Val) is located at the C-terminal loop of α3 (Fig. 3a). Residues 113–121 are located in the α_1_ helix known to participate in protein–protein interactions in RRM proteins [28].

Furthermore, the RRM domain interacts with COMPASS component WDR82 [5]. Thus, substitution of this 9-residue stretch could severely compromise the RRM fold and its interactions. p.(Met170Thr) and p.(Gly195Val) could affect substrate recognition of RRM because both residues are involved in RNA binding [29]. p.(Thr281Ile) and p.(Thr318Met) are located downstream of the RRM, in a disordered serine, threonine and proline-rich region containing numerous predicted phosphorylation sites [25]. Hence, p.(Thr281Ile) and p.(Thr318Met) might affect the phosphorylation landscape of this region. Substitutions p.(Arg429Trp), p.(Pro545Arg), p.(Pro698Ser), p.(Pro793arg), p.(Arg927His), p.(Arg982Gln), p.(Ala1010Val), p.(Ala1129Val), p.(Pro1328Ser), and p.(Arg1424Gln) are all located in the middle, largely disordered region of SETD1B. The middle portions of Setd1 proteins are divergent [1], suggesting they may have a role in differential genomic targeting of COMPASS through interaction with different targeting proteins. This role might be affected by the mostly nonconservative nature of these substitutions. p.(Ala1129Val), however, is predicted to introduce a noncanonical 5’ splice donor site at nucleotide position c.3384, which would result in a truncated protein p.(Ala1129fs) with eliminated SET catalytic domains. p.(Arg1748Cys) is located in the WIN motif (Fig. 3a) and expected to significantly decrease interaction between SETD1B and WDR5, which is essential for COMPASS assembly and SETD1B participation in H3K4 methylation [6]. Substitutions p.(Arg1792Trp), p.(Arg1825Pro), and p.(Lys1827Arg) are located at the interface with the nucleosome (Fig. 3a) and therefore likely affect interaction with histones and complex stability. Variants p.(Ala1901Val), p.(Ala1901Glu), p.(Tyr1941fs), and p.(Glu1948Lys) are located in the catalytic SET domain (Fig. 3a). Ala1901 is situated in a loop that is part of the SAdenosyl methionine (SAM) substrate-binding pocket, but is facing away toward an opposing β-strand that is part of the structural core of the SET domain. The substitution of alanine by the larger and negatively charged glutamic acid would create a large stress on the core of the SET domain and potentially disrupt the structural frame maintaining the SAM substrate-binding site and interactions with the adjacent subunits of the complex, whereas alanine to valine substitution introduces a small physicochemical difference which is likely to create some disturbance. p.(Tyr1941fs) would extend the protein, altering the SET domain and post-SET region that are involved in catalysis and cofactor binding, thus likely rendering SETD1B inactive (Fig. 3a). This C-terminal segment is highly conserved [24]. It covers a substantial portion of the binding pocket for histone H3 and the SAM substrate (including the SAM-binding Tyr1943), and three cysteine residues that together with Arg1962 coordinate a zinc atom. Glu1948 is located in a loop adjacent to the histone H3 binding site and, when superimposed to the yeast COMPASS EM structure (PDB:6ven), it is found to be close to the DNA binding surface between Set1 and Bre2 (homolog of ASH2) (Fig. 3a). The replacement of the glutamic acid by a lysine changes the charge of that side chain and could affect interactions of this region.

### Functional evaluation of selected *SETD1B* variants

Based on the structural modeling, seven variants in different regions of SETD1B were selected for in vitro studies: p.(His10Gln) and p.(Glu94Asp) N-terminal of RRM; p.(Asn113_Asp121delins9) in RRM; p.(Thr318Met) C-terminal of RRM; and p.(Ala1901Val), p.(Ala1901Glu), and p.(Glu1948Lys) in the catalytic SET domain.

First, stability of SETD1B in cells was evaluated by western blotting of wild-type and variant SETD1B overexpressed in HEK293 cells (Fig. 3b, Supplementary Fig. S4A). No significant differences in protein levels were observed, suggesting that the evaluated variants do not affect protein stability. Genomic targeting of SETD1B might depend on the central region and the catalytic domain, whereas RRM could reinforce chromatin binding [1, 30], resulting in distribution in the nucleus and not in the nucleoli. Therefore, SETD1B nuclear distribution of wild type and variant was assessed by immunofluorescence of transiently transfected HEK293 cells. Overexpressed FLAG-SETD1B was detected in the cytoplasm and nucleus. Nuclear localization patterns of SETD1B remained similar between wild type and variants, except for p.(Asn113_Asp121delins9), which failed to localize to the nucleus (Fig. 3c). Exclusion from the nucleus correlates with an inability to bind chromatin, resulting in loss of function of this variant. As suggested by structural modeling, Glu1948 could be involved in interaction with COMPASS subunit ASH2. Co-transfection and immunostaining were performed to evaluate colocalization (Fig. 3d). Both overexpressed SETD1B and ASH2 were detected in the nucleus and cytoplasm of transfected HEK293 cells, with a higher colocalization correlation for wild type compared to p.(Glu1948Lys) (Pearson’s correlation value of 0.5 and 0.3 respectively). To evaluate the effect of p.(Ala1901Val), p.(Ala1901-Glu), and p.(Glu1948Lys), protein stability and ligand binding were evaluated using thermal shift analysis of the catalytic domain (Fig. 3e). After GST-tagged SETD1B SET domain expression of wild type and variants, melting temperature (*T_m_*) was compared (Fig. 3e, left panel; Supplementary Fig. S4C, D). The *T_m_* of p.(Glu1948Lys) was 1.2 °C higher compared to wild type, which indicates that this substitution increases stability of the SET domain, which can result in disturbance of interactions within COMPASS, perhaps at the interface between SETD1B, the nucleosome, and the ASH2 subunit, as suggested by colocalization analysis of this variant with ASH2 subunit (Fig. 3d). Substitutions p.(Ala1901Val), p.(Ala1901Glu) resulted in a 0.3 °C negative and positive shift of *T_m_* respectively, suggesting that these substitutions have minor effects on thermal stability and thus on conformation of the SET domain. However, since these substitutions are predicted to influence interactions between SETD1B and the SAM substrate, the effect on *T_m_* in presence of SAM was evaluated (Fig. 3e, right panel). Generally, substrate-binding stabilizes proteins resulting in an increased *T_m_*, and indeed a mean *T_m_* increase of 0.3 °C was observed for wild type. The *T_m_* changes of the control GST-protein remained < 0.1 °C, suggesting no contribution of GST tag to the SAM interactions. The increase of 0.17 °C *T_m_* for both p.(Ala1901Val) and p.(Ala1901Glu) indicates no significant effect on SAM interaction.

A specific DNA methylation profile (episignature) for individuals with heterozygous loss-of-function pathogenic *SETD1B* variants has been described [8]. We performed episignature analysis for nine individuals (individuals 3, 4, 5, 7, 18, 19, 20, 31, 33), and the parents of individual 3 (Fig. 3f–g, Supplementary Fig. S4F). Individuals 5 (p.[Phe95*]), 7 (p.[Asn113_Asp121delins9]), 20 (p.[Ala1129Val]), 31 (p.[Ala1901Glu], and 33 (p.[Glu1948Lys]) showed the previously established SETD1B episignature; individual 18 (p.[Arg982Gln]) showed an inconclusive result, whereas individuals 3 (p.[His10Gln];[Arg927His], nor his parents 3.1 and 3.2), 4 (p.[Glu94Asp]);([Pro1328Ser]), and 19 (p.[Ala1010Val]) did not show the episignature associated with heterozygous loss-of-function *SETD1B* variants.

Taken together, through structural modeling and functional analyses we provide evidence for reduced function and therefore pathogenicity of p.(Phe95*), p.(Asn113_Asp121delins9), p.(Ala1129Val), p.(Ala1901Glu), and p.(Glu1948Lys), whereas functional consequences and clinical significance remains uncertain for p.(Thr318Met), p.(Arg982Gln), p.(Ala1010Val), p.(Ala1901Val), p.([His10Gln]); ([Arg927His]), and p.([Glu94Asp]);([Pro1328Ser]).

## Discussion

We report on the molecular and phenotypic spectrum of 36 individuals with sequence variants in *SETD1B*, representing the largest cohort reported to date. Previous work suggested a possible gain-of-function effect of pathogenic variants in *SETD1B* [14]; however, further reports [8, 12, 13, 15–19], including this work, point toward a loss-of-function mechanism. Clinical features of our cohort compared to previously reported individuals with a (likely) pathogenic *SETD1B* variant [8, 12–15] are provided in Table 2.

The emerging phenotype of *SETD1B*-associated disorder consists of global developmental delay, language delay including regression, intellectual disability, autism, and epilepsy. Other often observed neurobehavioral issues include hyperactivity, anxiety, anger, or aggressive behavior, and sleep disturbance. Importantly, in most cases, developmental delay predates seizure onset, and eight individuals (up to 16 years old) are seizure-free. This indicates that SETD1B dysfunction severely impacts physiological neurodevelopment even in the absence of epilepsy, suggesting the condition is a developmental encephalopathy, with or without epilepsy. Previously alterations of *SETD1B* were mainly associated with myoclonic absences [13] and predominantly refractory epilepsy. Although myoclonic absence seizures were often observed in our cohort—confirming this association—other seizure types were regularly encountered at onset, including focal or generalized tonic–clonic seizures. Epilepsy was well or partially controlled in most cases, with 7/26 (27%) remaining refractory to treatment. Brain imaging was unremarkable in most cases and observed abnormalities were without a consistent phenotype. Our cohort identifies a number of mild but consistent dysmorphisms in 30 individuals, including a prominent rounded nasal tip and bulbous nose, high anterior hairline, a thin upper lip, mild ear dysmorphisms, deep-set eyes, and mild hand abnormalities including tapering fingers, brachydactyly, small hands, and nail hypoplasia. Finally, previous work reported potential susceptibility to malignancy in SETD1B-related disorder [12]. Malignancies were not identified in our cohort, although this remains important for follow-up given the relatively young age of the cohort.

To identify possible genotype–phenotype correlation, a severity score was calculated for each individual in our cohort based on clinical features (Supplementary Methods). No association could be identified between the clinical severity score and the effect or location of the corresponding *SETD1B* variant (Supplementary Fig. S5). Intriguingly, there is a significant overrepresentation of males in both our cohort and in literature, with a total of 36 males and 16 females with *SETD1B* sequence variants reported (binominal test two-tailed *p* = 0.008) (Supplementary Methods). The reason for this remains unclear. Incidence of hypotonia and seizures did not differ between males and females in our cohort (hypotonia respectively 12/24, 50% and 4/11, 36%; seizures respectively 19/24, 79% and 9/12, 75%), and seizure onset was similar (respectively range and median years 0–12, 3 and 0–11, 2). Behavioral issues were seen more often in males than females (autistic behavior respectively 19/24, 79% and 5/12, 42%; hyperactivity respectively 10/23, 43% and 3/11, 27%; anxiety respectively 9/23, 39% and 2/12, 17%; aggression respectively 9/23, 39% and 2/12, 17%; sleep disturbance respectively 8/20, 40% and 2/12, 17%), although differences were not significant between both sexes. The clinical severity score is significantly lower in females compared to males, especially when considering behavioral features as a group (Supplementary Fig. S5). It is thus possible that females present with a milder phenotype that may not prompt medical evaluation. However, ascertainment bias for the neurodevelopmental phenotype could also contribute to the male predominance. Nevertheless, it is tempting to speculate that sex-linked traits could affect susceptibility to clinical penetrance and spectrum of *SETD1B* variants, as female-protective effects have been proposed for other neurodevelopmental disorders [31, 32].

We report four males from three families with biallelic variants in *SETD1B*, in which variants were inherited from unaffected parents. The two consanguineous siblings (individuals 11 and 12) share, besides the homozygous VUS in *SETD1B*, also homozygous VUS in *NBAS* (associated with immune defects) and *NOS1* (associated with achalasia). If disease causing, these variants could explain parts of the phenotypes of these individuals, but not their neurological findings. For both individuals, as well as for the other two individuals with biallelic *SETD1B* VUS, no alternative candidate variants were identified. Pathogenicity of the biallelic variants could not be experimentally proven by in vitro assays for p. (His10Gln) p.(Glu94Asp) and p.(Thr318Met), nor did p.([His10Gln]); ([Arg927His]) and p.([Glu94Asp]);([Pro1328Ser]) show the episignature previously associated with heterozygous *SETD1B* loss-of-function variants. However, this does not exclude the involvement of these variants in yet unknown SETD1B functions. Given that the phenotype of these individuals is similar to the heterozygous individuals (Table 2), and complete absence of SETD1B is lethal in several species [10, 33, 34], we speculate that the combined action of both alleles in biallelic cases results in a phenotype similar to that observed in heterozygous cases by reducing the remaining SETD1B activity below a required threshold. A small subset of genes that typically harbor de novo variants has already been associated with recessive inheritance [35]. Further investigations remain necessary to establish causality of these variants, and the possibility of recessive inheritance of the SETD1B-related disorder.

*SETD1B* adds to a growing list of chromatin modifying genes implicated in neurodevelopmental disorders. SETD1B is one of the six H3K4 methyltransferases present in mammals, and remarkably loss of function of each is associated with human disease (*KMT2A*: Wiedemann–Steiner syndrome [OMIM 605130]; *KMT2B*: early-onset dystonia [OMIM 617284]; *KMT2C*: Kleefstra syndrome type 2 [OMIM 617768]; *KMT2D*: Kabuki syndrome [OMIM 147920]), with the latest additions to this list being *SETD1A* and *SETD1B* (also known as *KMT2F* and *KMT2G*, respectively). SETD1B is paralogous to SETD1A (derived from the orthologue Set1) and both associate with the same noncatalytic COMPASS components. SETD1A and SETD1B, however, show nonoverlapping localization within the nucleus and thus likely make nonredundant contributions to epigenetic control of chromatin structure and gene regulation [1]. This might explain why both *SETD1A* and *SETD1B* knockout mice are embryonically lethal, albeit at different developmental stages [33]. Also, in adult mice, *SETD1B* knockout is lethal and provokes severe defects in hematopoiesis [34]. Heterozygous pathogenic variants in *SETD1A* have been described in individuals with developmental delay, intellectual disability, subtle facial dysmorphisms, and behavioral and psychiatric problems [36] (OMIM 619056). Interestingly, despite the anticipated nonredundant contributions of SETD1A and SETD1B to epigenetic control, the clinical phenotype of both related disorders shares many similarities [36]. These include global developmental delay with motor and language delay, intellectual disability, and behavioral abnormalities. *SETD1A* variants have also been found in schizophrenia cohorts [36] and mouse models support SETD1A involvement in schizophrenia [37]. One likely pathogenic *SETD1B* variant without clinical information was identified in a schizophrenia cohort [38], but psychosis was not reported in our *SETD1B* cohort. Given the relatively young age of the cohort, this will be an important point for follow-up. Noticeable differences between both syndromes are the incidence of epilepsy, which is more common for *SETD1B* (20% in *SETD1A* [36], 78% in this cohort), and the absence of a male predominance for *SETD1A* (9 males of 19 cases [36, 39]).

Germline mutants of *Set1*, the orthologue of *SETD1A* and *SETD1B* in *Drosophila melanogaster*, are embryonically lethal [10], whereas postmitotic neuronal knockdown shows that *Set1* is required for memory in flies, suggesting a role in postdevelopment neuronal function [36]. In *Caenorhabditis elegans*, the SETD1A/SETD1B orthologue Set-2 is important for transcription of neuronal genes, axon guidance, and neuronal functions [9], further underscoring the importance of both SETD1A and SETD1B for neural function. Interestingly, whereas we found multiple missense variants in the functional domain of SETD1B (RRM, N-SET, SET), in SETD1A only one missense variant is reported within a functional domain (post-SET). Finally, of the 23 missense variants found in SETD1B, 17 are in regions that are homologous in SETD1A. Of note, p.(Arg982Gln) in the disordered region is at a homologous position in SETD1A previously described in a patient with early-onset epilepsy (NM_014712.2(SETD1A):c.2737C>T, p.(Arg913cys]) [39]. It will be interesting to decipher the downstream epigenetic alterations causative for the resulting overlaps and differences in phenotype between both syndromes.

## Supplementary Material

The online version contains supplementary material available at https://doi.org/10.1038/s41436-021-01246-2.

Supplementary material combined

## Figures and Tables

**Table 1:** Overview phenotypic features.

Case number		1	2	3	4	5	6	7	8	9	10	11	12	13	14	15	16	17	18	19	20	21	22	23	24	25	26	27	28	29	30	31	32	33	34	35	36
Age at last evaluation		8y	10y	21y	11y	7y	8y	30y	21y	8y	19y	14y	11y	5y 3mo	2y 3mo	19y	1y	16y	13y	3y	7y	4y	3y 6mo	6y 3mo	14y	7y 4mo	13y	13y 4mo	5y 11mo	15y 5mo	7y	22y	15y	44y	3y	8y	2y 6mo
Sex		Male	Female	Male	Male	Female	Male	Male	Female	Male	Male	Male	Male	Male	Female	Male	Female	Female	Female	Male	Male	Female	Male	Male	Male	Male	Male	Female	Female	Female	Male	Male	Male	Male	Male	Male	Female
SETD1B variant		c.22dup, p.(His8fs)	c.22dup, p.(His8fs)	c.30C>A, p.(His10Gln); c.2780G>A, p.(Arg927His)	c.282G>C, p.(Glu94Asp); c.3982C>T, p.(Pro1328Ser)	c.284_286delinsA, p.(Phe95*)	c.288del, p.(Tyr96*)	c.337_363inv, p.(Asn113_Asp121delins9)	c.509T>C, p.(Met170Thr)	c.584G>T, p.(Gly195Val)	c.842C>T, p.(Thr281Ile)	c.953C>T, p.(Thr318Met)	c.953C>T, p.(Thr318Met)	c.1234del, p.(Glu412fs)	c.1285C>T, p.(Arg429Trp)	c.1634C>G, p.(Pro545Arg)	c.2092C>T, p.(Pro698Ser)	c.2378C>G, p.(Pro793Arg)	c.2945G>A, p.(Arg982Gln)	c.3029C>T, p.(Ala1010Val)	c.3386C>T, p.(Ala1129Val)	c.3985C>T, p.(Arg1329*)	c.4271G>A, p.(Arg1424Gln)	c.4570C>T, p.(Arg1524*)	c.4996C>T, p.(Gln1666*)	c.5184_5185del, p.(Ala1730*)	c.5242C>T, p.(Arg1748Cys)	c.5374C>T, p.(Arg1792Trp)	c.5474G>C, p.(Arg1825Pro)	c.5480A>G, p.(Lys1827Arg)	c.5702C>T, p.(Ala1901Val)	c.5702C>A, p.(Ala1901Glu)	c.5820_5826del, p.(Tyr1941fs)	c.5842G>A, p.(Glu1948Lys)	c.5842G>A, p.(Glu1948Lys)	c.5842G>A, p.(Glu1948Lys)	c.5842G>A, p.(Glu1948Lys)
Class (ACMG/AMP)		5 (Pathogenic)	5 (Pathogenic)	3 (Uncertain significance); 3 (Uncertain significance)	3 (Uncertain significance); 3 (Uncertain significance)	5 (Pathogenic)	5 (Pathogenic)	5 (Pathogenic)	4 (Likely pathogenic)	4 (Likely pathogenic)	3 (Uncertain significance)	3 (Uncertain significance)	3 (Uncertain significance)	5 (Pathogenic)	4 (Likely pathogenic)	3 (Uncertain significance)	4 (Likely pathogenic)	4 (Likely pathogenic)	3 (Uncertain significance)	3 (Uncertain significance)	5 (Pathogenic)	5 (Pathogenic)	4 (Likely pathogenic)	5 (Pathogenic)	5 (Pathogenic)	5 (Pathogenic)	4 (Likely pathogenic)	4 (Likely pathogenic)	4 (Likely pathogenic)	4 (Likely pathogenic)	5 (Pathogenic)	5 (Pathogenic)	5 (Pathogenic)	5 (Pathogenic)	5 (Pathogenic)	5 (Pathogenic)	5 (Pathogenic)	
Zygosity		Heterozygous	Heterozygous	Compound heterozygous	Compound heterozygous	Heterozygous	Heterozygous	Heterozygous	Heterozygous	Heterozygous	Heterozygous	Homozygous	Homozygous	Heterozygous	Heterozygous	Heterozygous	Heterozygous	Heterozygous	Heterozygous	Heterozygous	Heterozygous	Heterozygous	Heterozygous	Heterozygous	Heterozygous	Heterozygous	Heterozygous	Heterozygous	Heterozygous	Heterozygous	Heterozygous	Heterozygous	Heterozygous	Heterozygous	Heterozygous	Heterozygous	Heterozygous	
Inheritance		De novo	De novo	One paternal and one maternal (both unaffected)	One paternal and one maternal (both unaffected)	De novo	De novo	De novo	De novo	De novo	Paternal (unaffected)	Parents are heterozygous (both unaffected)	Parents are heterozygous (both unaffected)	Maternal (affected)	De novo	Unknown (patient adopted)	De novo	De novo	Unknown (patient adopted)	De novo	De novo	De novo	De novo	De novo	De novo	De novo	De novo	De novo	De novo	De novo	De novo	De novo	De novo	De novo	De novo	De novo	De novo	
Seizure	Seizures	Yes	Yes	Yes	Yes	No	No	Yes	Yes	No	Yes	Yes	Yes	Yes	Yes	Yes	Yes	No	Yes	Yes	Yes	Yes	Yes	No	Yes	Yes	Yes	Yes	Yes	Yes	No	Yes	Yes	Yes	No	Yes	No	
Seizure onset (years)		6	4	2	0 (day 1)			1	3		0 (neonatal)	10	5	4	2	6	0 (3 mo)		1	1	2	2	3		6	3	9 (6)	0 (6mo)	4	11		12	1	3		2		
Seizure type at onset (course of seizures)		Myoclonic absence (Generalized tonic clonic)	Focal	Focal (Lennox-Gastaut)	Tonic and apnea (Focal)			Myoclonic, generalized and absence	Atypical absence (Generalized tonic clonic)		Infantile spasms	Generalized tonic clonic (status epilepticus, absences)	Generalized tonic clonic (simple partial)	Atonic	Focal and generalized	Absence (staring spells, fatigue)	Focal		Atonic (eyelid myoclonia, absence, tonic)	N/A	Convulsions during fever (generalized epilepsy)	Eyelid myoclonia (Myoclonic)	Absence with eyelid myoclonia		Absence and focal	Myoclonic (Eyelid myoclonia)	Absence	Absence (Generalized tonic-clonic)	Absence	Absence (Drop, nocturnal tonic-clonic)		Myoclonic	Atypical absence, potentially myoclonic absence (Lennox-Gastaut)	Generalized		Absence (Absence-atonic/myoclonic, generalized tonic-clonic)		
Frequency of seizure (prior to treatment)		Several per day	Infrequent to almost daily	Very frequent	N/A			Several per day	30-40 per day		3-5 per week	N/A	N/A	N/A	2 in 40 days	1 per month	2 in 1 day		50-100 per day	Sporadic	20 per day	70-200 per day	Daily		>1 (30-40) per day	7-8 per day	10-30 per day	Multiple per day	NA	N/A		10-15 per day	Every other day	N/A		30-40 per day		
Response to treatment		Yes (LVT)	Yes (partly) (VPA, LVT)	No (VPA, Lamotrigine)	Yes (VPA/oxcarbazepine, clobazam)			Yes (at 8y treatment stopped, insult-free)	No		No	Yes (carbamazepine, fenitoin and VPA)	Yes (fenitoin and VPA)	Yes	Yes (phenobarbital)	Yes (LVT and VPA)	N/A		No	N/A	Yes (VPA)	No	Yes (VPA)		Yes, partly (VNS and Cannabidiol)	Yes (VPA, Clonazepam)	No	Yes, partly (Ethosuximide, Topiramate)	Yes	Yes (Lamotrigine)		Yes, partly	No	Yes (LVT)		Yes, partly (Ethosuxamide; VPA, clobazam, Lamotrigine, Diazepam)		
Ketogenic diet tried		No	No	No	No			N/A	Yes		Yes	No	No	No	No	No	No		Yes (modified Atkins)	N/A	No	Yes (modified Atkins)	No		Yes	N/A	No	No	No	No		No	No	No		No		
Initial EEG		Interictal bursts of polyspike and wave, ictal EEG captured brief myoclonic absence seizure 4hz GPSW	Runs of high amplitude epileptiform discharges noted over F3, C3, P3 and Pz	awake: occasional spikes from both hemispheres, especially centroparietal; asleep: abundant multifocal spikes, especially from centroparietal areas (right>left). In EEG background no reaction to eye opening, no sleep phenomena	Ictal EEG (at 11 months): no epileptic discharges during nystagmus. Asymmetrical EEG			N/A	Generalized 2.5-3Hz spike and wave with photosensitivity		Hypsarrhythmia initially, then multifocal independent spike discharges bilaterally, later focal spike and polyspike waves	N/A	N/A	1. Frequent bifrontally maximal generalized spike/polyspike-wave activity (Max. 2 sec.) 2. Borderline generalized theta slowing for age	Normal	Right centrotemporal spikes	Normal		N/A	N/A	EEG at 3y: normal background pattern with 6x short (ca 2 sec) generalized epileptic activity, maximum activity bilateral frontal. At that time starring and head nodding. In addition, isolated focal discharges intermitting frontotemporal left and right, indicating a generalized epilepsy syndrome not further specified.	Frequent (>20) myoclonic seizures (2-4 Hz generalized spike and waves clinically correlating with sudden brief generalized full body jerk, lasting for 1-2 sec.); Borderline mild diffuse background slowing for age - Occasional independent bilateral frontal and bilateral posterior temporal spikes - Frequent, very brief (0.5-3 sec) bursts of generalized spike-wave and polyspike-wave complexes at ~2.5-5 Hz, often with a posterior onset, occurring up to 15-20 times per hour	Ictal EEG: bilateral fronto-temporal and generalized spike-wave discharges		Frequent generalized polyspike-wave discharges associated with unresponsiveness as well as multifocal epileptiform discharges	Interictal: large amount of generalized 2-4 Hz spike-slow or poly-spike-slow waves burst or paroxysm with or without clinical seizure. Ictal: generalized medium to very-high voltage of 2-4 Hz spike-slow or poly-spike-slow waves burst or paroxysm with clinical seizure manifested as blinking with or without limb saking	Very frequent generalized polyspike and polyspike-wave complexes, 2-3 per sec., both spontaneously as upon closure of eyes. Sometimes focal left frontotemporal component. In general fitting to an absence epilepsy with atypical features	Abrupt onset generalized spike-wave activity, 3-3.5 Hz	N/A	Abnormal		N/A	Interictal: focal epileptiform discharges in left temporal and parietal areas. As the patient became drowsy, high amplitude discharges which were left maximal but with diffuse field in long run of variable frequency were seen but no clear ictal pattern	N/A		Recurrent bursts of polyspike and wave discharges in generalized distribution, greater prominence over left hemisphere with independent epileptiform discharge activity also on the right. Thought to be having absence seizures from generalized epilepsy		
Course of EEG		N/A	At 4-5years of age- a few transient burst of poly-spike-wave discharges in sleep, none in wake. At 6 years of age: runs of high amplitude discharges over wide area of left hemisphere. At 10 years left localized discharges in sleep and generalized bursts on HV. Background activities remain normal	Findings were similar in repeated recordings	Interictal EEG (7 years of age): no signs of epileptic discharges, neither during sleep			N/A	Serial records suggest pattern remained unchanted; recent EEG shows 3-4Hx spike and wave, and background slowing		Most recent EEG at 13 years showed no epileptiform activity but he has symmetric spindle stage sleep pattern	N/A	N/A	The EEG showed severe bifrontal dominant spike waves	The left parietal and occipital start, followed by large slow waves in various areas on the right, followed by spike wave and slow wave, left	Initially focal, now thought to be generalized			at 13 years, 7 months: Abnormal EEG due to: 1. Generalized seizures consistent with eyelid myoclonia 2. Generalized epileptiform discharges during the awake and sleep states 3. Events of concern (headache and dizziness) captured without EEG correlate 4. Normal awake and asleep background at 13 years, 6 months: Abnormal EEG due to: 1- Photic induced myoclonic seizures 2- Eyelid myoclonia without absence seizures 3- Background: Normal for age. at 13 years, 2 months: Abnormal EEG due to: 1- Eyelid closure induced and photosensitive eyelid myoclonia with and without absences 2- Generalized and focal epileptiform discharges. 3- Background: Mild diffuse slowing. at 12 years, 1 months: Abnormal (awake, drowsy) 1-Polyspike/spike and wave, generalized 10/16/19: Abnormal EEG due to: 1. Generalized seizures consistent with eyelid myoclonia 2. Generalized epileptiform discharges during the awake and sleep states 3. Events of concern (headache and dizziness) captured without EEG correlate 4. Normal awake and asleep background 9/23/2019: Abnormal EEG due to: 1- Photic induced myoclonic seizures 2- Eyelid myoclonia without absence seizures 3- Background: Normal for age. 5/14/2019 Abnormal EEG due to: 1- Eyelid closure induced and photosensitive eyelid myoclonia with and without absences 2- Generalized and focal epileptiform discharges. 3- Background: Mild diffuse slowing. 4/4/2018 Abnormal (awake, drowsy) 1-Polyspike/spike and wave, generalized	N/A	N/A	-Frequent generalized 3-3.5 Hz spike and polyspike wave discharges -Frequent focal spikes in the left parieto-occipital region more so than right frontal region -Frequent brief absence seizures with eyelid myoclonia -Mild diffuse slowing for age; - Borderline mild diffuse background slowing for age - Occasional independent bilateral frontal and bilateral posterior temporal spikes - Frequent, very brief (0.5-3 seconds) bursts of generalized spike-wave and polyspike-wave complexes at ~2.5-5 Hz, often with a posterior onset, occurring up to 15-20 times per hour; third 8/20: - Frequent (at least 20) myoclonic seizures (2-4 Hz generalized spike and waves clinically correlating with sudden brief generalized full body jerk, lasting for 1-2 second) - Intermittent rhythmic central theta activity without any clinical change V15	Generalized spike-wave discharges		Most recent EEG at 11 years: frequent bursts of irregularly formed, generalized bi-frontally predominant spikes	Interictal: large amount of generalized medium to very-high voltage 3-4.5 Hz spike-slow, poly-spike-slow or slow waves sporadic discharge or paroxysm, sometimes focus on frontotemporal area, Ictal: generalized 3-4.5 Hz spike-slow or poly-spike-slow waves paroxysm with clinical seizure manifested as blinking or limb saking one times	Last EEG: epileptiform paroxysms (temporal and frontal) in >80% of the registration. Alternating generalized and multifocal	Last EEG at age 9 years and 9 months. On going to sleep: large amount of spike wave activity 4-5 Hz. Two similar bursts during day. Interictal - more spike-wave activity than previous EEG	N/A	N/A		N/A	N/A	N/A		Last vEEG at age of 3 years, 9 month showed mild encephalopathy with frequent paroxysms of generalized bisynchronous 3 to 3-1/2 cycles per second spike and slow wave discharges. These were at the time lateralized towards the left or right hemisphere indicative of a generalized epilepsy syndrome		
Brain MRI (age)		Abnormalities (7y)	Unremarkable (7y)	Unremarkable	Abnormalities (6y)	Unremarkable	Unremarkable (4y)	Unremarkable (4y, 7y)	None	None	Abnormalities (7y)	Unremarkable	Unremarkable	Unremarkable	Unremarkable	Unremarkable	Abnormalities	Unremarkable	Unremarkable (9y)	Abnormalities	Unremarkable	Abnormalities (4y)	Unremarkable	Unremarkable (6y)	Unremarkable (12y)	Unremarkable (4y)	Unremarkable	Unremarkable (incidental finding)	Unremarkable	Unremarkable	Abnormalities	None	Abnormalities	None	Unremarkable	Unremarkable (3y)	None	
Developmental delay	Development	Yes	Yes	Yes	Yes (regressed)	Yes	Yes	Yes	Yes (regressed)	Yes	Yes	Yes (regressed)	Yes (regressed)	Yes (regressed)	No	Yes	No	Yes	Yes (regressed)	Yes	Yes	Yes	Yes	Yes	Yes	Yes	Yes	Yes	Yes	Yes	Yes (regressed)	Yes	Yes (regressed)	Yes	Yes (regressed)	Yes	Yes	
Motor development (age at first walking)		Delayed (1y 7mo)	Delayed (4y)	Delayed (3y)	Delayed (4y)	Delayed (2y 9mo)	Delayed (1y 6mo)	Delayed (1y 8mo)	Delayed (N/A)	Delayed (1y 7mo)	Delayed (no walking)	Delayed (3y)	Delayed (1y 6mo)	Delayed (1y 1mo)	Normal (1y 4mo)	Delayed (1y 1mo)	Normal (1y)	Delayed (1y 6mo)	Normal (1y)	Delayed (2y 6mo)	Delayed (1y 6mo)	Delayed (1y 2mo)	Delayed (3y)	Delayed (1y 6mo)	Delayed (1y 5mo)	Delayed (1y)	Normal (1y)	Delayed (1y 8mo)	Delayed (2y)	Delayed (4y 6mo)	Delayed (1y 6mo)	Delayed (>2y 6mo)	Delayed (1y 8mo)	Delayed (N/A)	Delayed (1y 7mo)	Delayed (1y 2mo)	Delayed (2y 5mo with support)	
Hypotonia		No	No	Yes	No	Yes	No	Yes (infant)	N/A	No	Yes (infant)	No	No	No	No	No	No	Yes	No	No	Yes	No	Yes	Yes (mild)	Yes	No	No	Yes (infant/child)	No	Yes	Yes	Yes (neonatal)	No	Yes	Yes	Yes	No	
Language development (age at first word)		Delayed (2y 1mo)	Delayed (3y)	Delayed (some words)	Delayed (only sounds at 11y)	Delayed (2y)	Delayed (2y)	Delayed (N/A) (speech therapy)	Delayed (3y)	Delayed (2y 6mo)	Delayed (non-verbal)	Delayed (no phrases)	Delayed (no phrases)	Delayed (1y)	Normal (10mo)	Delayed (1y 6mo)	Normal (1y)	Delayed (N/A)	Delayed (limited speech)	Normal (1y 3mo)	Delayed (2y)	Delayed (1y)	Delayed (non-verbal)	Delayed (9 mo)	Delayed (1y 3mo)	Delayed (6mo)	Delayed (N/A)	Delayed (2y) (short sentences)	Delayed (N/A)	Delayed (2y)	Delayed (1y 6mo)	Delayed (3y)	Delayed (2y 6mo)	Delayed (N/A)	Delayed (non-verbal)	Delayed (2y)	Delayed (non-verbal)	
Intellectual disability (tested IQ)		Mild (57)	Moderate (45)	Severe (N/A)	Severe (N/A)	Yes (N/A)	Mild (70)	Mild (77)	Yes (N/A)	Moderate (N/A)	Severe (N/A)	Yes (N/A)	Yes (N/A)	Suspected (N/A)	No (N/A)	Mild (62)	No (N/A)	Yes (N/A)	Moderate (41)	N/A	Mild (60)	N/A	Severe (N/A)	Moderate (N/A)	Mild (69)	No (N/A)	Mild (60-70)	Mild (N/A)	N/A	Moderate (47)	No	Moderate (48-52)	Yes (N/A)	Moderate (N/A)	N/A	Mild (N/A)	Moderate (N/A)	4
Autism / autistic behavior	Behavior	Yes (but does not meet ASD criteria)	No	No	Yes	No	Yes	Yes	Yes	Yes	Yes	Yes	Yes	Yes	No	Yes	No	No	Yes	No	Yes	No	No	No	Yes	No	Yes	Yes (but does not meet ASD criteria)	No	Yes	Yes	Yes	Yes	Yes	Yes	Yes	Yes	
Hyperactive		No	No	Yes	Yes	No	Yes	Yes	No	No	No	No	No	Yes	No	Yes	No	No	Yes	No	No	No	No	Yes	No	No	N/A	Yes	No	N/A	Yes	Yes	No	No	Yes	No	Yes	
Anxiety		No	No	No	No	No	Yes	No	No	Yes	No	Yes	Yes	No	No	Yes	No	No	Yes	No	Yes	No	No	No	Yes	No	N/A	No	No	Yes	Yes	No	No	No	No	Yes	No	
Aggressive behavior		Yes (tantrums)	No (moody)	No	Yes (self-mutilation, pica)	No	No	Yes (as a child)	No	No	Yes (self-injurious)	No	No	Yes	No	No	No	No	No	Yes (biting during play)	No	No	No	No	Yes	No	N/A	Yes (at home)	No	Yes (when anxious)	No	Yes (tantrums, pica)	No	No	No	Yes (ODD symptoms)	No (does not play appropriately)	
Sleep disturbance		No	No	No	Yes (until 7y)	No	No	N/A	No	No	No	Yes	N/A	Yes (melatonin)	No	No	No	No	Yes (melatonin)	Yes	No	No	No	Yes	No	No	N/A	No	No	No	Yes	Yes	N/A	No	No	Yes	Yes	
Craniofacial	Dysmorphology	Deep set eyes, cupped helices, large ear lobes, short philtrum, chin dimple, brachycephaly	Sparse eyebrow, hypertelorism, prominent nasal tip	High anterior hairline, prominent nasal tip, thin upper lip, elongated head, bitemporal narrowing	High anterior hairline, cup-shaped ears, low-set ears, large earlobe, over-folded superior helices, bulbous/rounded nasal tip, sunken nasal root, flattened nasal bridge, bitemporal narrowing, frontal bossing	High anterior hairline, sparse eyebrow medially (wide in the middle), arched eyebrows, hypertelorism, low-set ears, preauric tag right ear, bulbous nose, broad nasal base, prominent rounded nasal tip, midface hypoplasia, large fontanel	Synophrys, slightly elongated palpebral fissures, large earlobes, bulbous nose, high arched and narrow pallatum	Slightly small ears, long face, microcephaly (-2 SD)	N/A	Narrow upslanting deep set palpebral fissures, epicanthal folds, fleshy (large) ear lobes, prominent rounded nasal tip, broad nasal root, full cheeks	Not dysmorphic	Low-set ears, uplifted large earlobe, macrognatia, round face	Low-set ears, uplifted large earlobe, macrognatia, round face	High anterior hairline, prominent eyebrows/eyelashes, straight downslanted eyebrows, downslanted palpebral fissures, bulbous/prominent nasal tip, prominent lips, course face, macrocephaly*	Not dysmorphic	Almond-shaped eyes, large ears, narrow-shaped/elongated head	Not dysmorphic.	Not dysmorphic	Widow's peak, anterior hairline regression, thick eyebrows, deep set eyes, rounded nasal tip, mild bulbous nose, thin upper lip, slightly smooth philtrum, thin thick vermilion, mild pointed chin	microcephaly (-2.5SD)	Upslant, epicanthus, upturned nose, bulbous tip, overfolded helix, long philtrum, small mouth, small chin, (mild) dolichocephaly	Not dysmorphic (no full examination performed)	High anterior hairline, mild arched eyebrows, downslanting palpebral fissures, epicanthal folds, mild large earlobe, flattened nasal bridge, large nasal root, thin upper lip, thick vermilion, mild sloping forehead, frontal bossing, plagiocephaly	Long face, hypertelorism	Low frontal hairline, synophrys, upslanting palpebral fissures, posterior helical pits (2 left 1 right), simplified helices, broad nasal tip (upslanting), thin upper lip, wide mouth, long tongue, bitemporal narrowing	Uplifted earlobe	Epicanthal folds, bulging fontanelle, macrocephaly (+2 SD)	Short palpebral fissures, anteverted nares, short nose	High anterior hairline, full eyebrows with synophrys, upslanting palpebral fissures, small everted low-set ears, over-folded superior helices, broad nasal base, round nasal tip, broad mouth with full lips, full cheeks, pointed chin, prominent forehead, square face, frontal bossing	High anterior hairline, sparse eyebrows, deep-set eyes, hypoplastic alae, rounded nasal tip, bulbous nose, thin upper lip, full cheeks	Deep-set eyes, small mouth	Frontal balding, hypoplastic alae nasi, long columnella, thin upper lip, high arched pallatum	Full cheeks	High anterior hairline, upslanted (mild) palpebral fissures, broad nasal base, large nose, pointed chin, elongated face and mandibula, slightly asymmetric face	Flattened nasal bridge, anteverted nares, thin upper lip, mild micrognathia	Uplifted large earlobe, narrow over-folded superior helices, narrow nasal base and root, prominent (round) nasal tip and columella, (mild) bulbous nose, thin upper lip, prominent chin, bitemporal narrowing	High anterior hairline, synophrys, deep set eyes, small palpebral fissures, ear tags (removed), overfolded superior helices, prominent nasal root and tip, thin upper lip, frontal bossing	
Ophthalmological			Myopia both eyes		Strabismus (eso- and exotropia)			Strabismus (mild)		Astigmatism	Cortical vision impairment, small unilateral cataract							Strabismus (squint)			N/A						Strabismus (operated)			Myopia, astigmatism	Astigmatism, refractive amblyopia			Amblyopia, strabismus		Ptosis		
Musculoskeletal		Tapering fingers		Short fingers/brachydactyly, sandal gap	Pes planus, Joint hypermobility	Brachydactyly, clubfeet, contractures elbows knees (likely due to oligohydramnios)		Small hands and feet		Small hands, Joint hypermobility	Scoliosis	Scoliosis, possible right fibula hemimelia	Scoliosis	Large hands/feet		Bulging disc			5th digit clinodactyly, brachydactyly, hyperextensible small joints		Slender fingers, slight tapering, pes planus							Short terminal phalanges, short 5th fingers with mild clinodactyly, joint hypermobility	Tapering fingers, sandal gaps, clinodactyly 4 and 5 digits	Tapering fingers, pes planus, kyphosis		Overfolding toes 3 and 4	tapered fingers, 5th finger clinodactyly, planovalgus deformity, talocalcaneal coalitions, dextroscoliosis, borderline osteopenia	Small hands and feet, scoliosis, kyphosis, pes cavus		Narrow hands, short 5th finger, mild pectus excavatum		
Dermatological			Hyperpigmented areas		Eczema			Very dry skin				Ecchymosis						Eczema	Eczema, hypopigmented area, café au lait spot (2-4 cm)	Eczema (rough skin)						Café au lait macules (<1 cm)					Eczema, facial aseptic granuloma (idiopathic)							
Other				Nail hypoplasia	Inverted nipples. Obstipation (Ileocecal invagination)	Feeding problems, dysplastic kidneys (transplanted), anterior placed anus, clubfeet, tethered cord, contractures.		Irritable bowel syndrome			Feeding problems. *concurrent Osteogenesis Imperfecta and paternal NPRL3 (3, VOUS)	Teeth cavities *concurrent Immune defects (NBAS) and Achalasia (NOS1)	Teeth cavities *concurrent Immune defects (NBAS) and Achalasia (NOS1)	*concurrent PTEN-related disorder		Hyperconvex deepset toenails		Reflux, bowel issues	Nail hypoplasia (hand and feet). Constipation	Transverse palmar creases	Cryptorchidism (operated)			Strength normal to slightly low	Small appearing teeth/gingival overgrowth	Inverted nipples	Inverted nipples, mild gynecomastia	Short fingernails. Constipation				Small nails. Impaired hearing	Low muscle bulk and thin extremities		Right transverse palmar crease, delayed tooth eruption. Vestibular dysfunction. Reflux, feeding problems.	Simple hyperconvex toenails, irregular teeth eruption		
Hypoglycemia	Other	Neonatal (GDM)	No	No	No	No	No	Neonatal (GDM)	No	No	Yes (on ketogenic diet)	N/A	N/A	No	No	No	Yes	No	N/A	No	N/A	No	No	Neonatal	No	No	Neonatal	No	Neonatal	No	No	N/A	No	No	Neonatal (GDM)	No	No	
Overweight/obesity		Yes	Yes	N/A	No	No	Yes	Yes (mainly truncal)	No	Yes	No	No	No	Yes	Yes	Yes	N/A	No	Yes (mainly truncal)	Yes	Yes	No	No	No	No	Yes	Yes	No	Yes	Yes	Yes	Yes	No	No	No	No	No	

A: aberrant, N: normal. NA not available. GDM Gestational diabetes mellitus

A aberrant, *ACMG/AMP* American College of Medical Genetics and Genomics/Association for Molecular Pathology, *ASD* autism spectrum disorder, *EEG* electroencephalogram, *GDM* gestational diabetes mellitus, *GPSW* generalized polyspike-wave, *HV* hyperventilation, *LVT* levetiracetam, *MRI* magnetic resonance image, *N* normal, *NA* not available, *ODD* oppositional defiant disorder, *vEEG* video EEG, *VNS* vagal nerve stimulation, *VPA* valproate, *VUS* variant of uncertain significance.

**Table 2:** Summary of main phenotypic features in this study cohort and in literature.

	This study	Literature	This study	This study
*Variant classification (ACMG/AMP)*	***(Likely) Pathogenic***	*(Likely) Pathogenic*	***Uncertain significance***	***Uncertain significance***
*Variant zygosity*	***Heterozygous***	*Heterozygous*	***Heterozygous***	***Bi-allelic***
*Sex (male)*	17/28 (61%)	8/11 (73%)	3/4 (75%)	4/4 (100%)
*Seizure*	20/28 (71%)	10/10 (100%)	4/4 (100%)	4/4 (100%)
*Seizure type at onset (generalized)*	16/20 (80%)	8/9 (89%)	1/1 (100%)	2/3 (66%)
*Response to treatment (yes/partly)*	15/19 (79%)	3/7 (43%)	1/3 (33%)	3/4 (75%)
*Developmental delay*	26/28 (93%)	11/11 (100%)	4/4 (100%)	4/4 (100%)
*Motor development*	25/28 (89%)	7/9 (78%)	3/4 (75%)	4/4 (100%)
*Language development*	26/28 (93%)	11/11 (100%)	3/4 (75%)	4/4 (100%)
*Intellectual disability*	21/25 (84%)	11/11 (100%)	3/3 (100%)	4/4 (100%)
*Autism / autistic behavior*	18/28 (64%)	7/11 (64%)	3/4 (75%)	3/4 (75%)
*Other behavioral issues*	15/27 (56%)	3/7 (43%)	4/4 (100%)	4/4 (100%)
*Sleep disturbance*	6/25 (24%)	N/A	2/4 (50%)	2/4 (50%)

As information on the different features was not always available for each individual, the denominator of the frequencies differs between the different clinical characteristics. **ACMG/AMP** American College of Medical Genetics and Genomics/Association for Molecular Pathology.

## Data Availability

The data that support the findings of this study are available from the corresponding author, with the exception of primary patient sequencing data, as they are derived from patient samples with unique variants that are impossible to guarantee anonymity for. Our institutional guidelines do not allow sharing these raw exome or genome sequencing data, as this is not part of the patient consent procedure.
