# Supplementary material for: Delineating the molecular and phenotypic spectrum of the SETD1B-related syndrome
Source: Genet Med. Author manuscript; Available in PMC 2021 Nov 3. (PMC8553606; doi:10.1038/s41436-021-01246-2)
Supplement: Supplementary material combined [file EMS133143-supplement-Supplementary__material_combined.pdf]

Supplementary Material:

Delineating the molecular and phenotypic spectrum of the *SETD1B*-related syndrome

Marjolein J.A. Weerts, PhD*, Kristina Lanko, PhD*, Francisco J. Guzmán-Vega, M.Sc, Adam Jackson, MB ChB, Reshmi Ramakrishnan, PhD, Kelly J. Cardona-Londoño, M.Sc., Karla A. Peña-Guerra, B.S., Yolande van Bever, MD, Barbara W. van Paassen, MD, Anneke Kievit, MD, PhD, Marjon van Slegtenhorst, PhD, Nicholas M. Allen, MB.BCh, MDRes, Caroline M. Kehoe, MB., BCh., Hannah K. Robinson, PhD, Lewis Pang, MSc, Selina H. Banu, MBBS, DCH, PhD, Mashaya Zaman, MBBS, Stephanie Efthymiou,MSc, Henry Houlden, MD, PhD, Irma Järvelä, MD, Leena Lauronen, MD, Tuomo Määttä, MD, Isabelle Schrauwen, PhD, Suzanne M Leal, PhD, Claudia A.L Ruivenkamp, PhD, Daniela Q.C.M. Barge-Schaapveld, MD, PhD, Cacha M.P.C.D. Peeters-Scholte, MD, PhD, Hamid Galehdari, PhD, Neda Mazaheri, PhD, Sanjay M Sisodiya, PhD FRCP, Victoria Harrison, MB ChB, Angela Sun, MD, Jenny Thies, CGC, Luis Alberto Pedroza,PhD, Yana Lara-Taranchenko, MD, Ivan K. Chinn, MD, James R. Lupski, MD, PhD, Alexandra Garza-Flores, MD, Jefferey McGlothlin, MD, Lin Yang, MD, Shaoping Huang, MD, Xiaodong Wang, PhD, Tamison Jewett, MD, Gretchen Rosso, MS, CGC, Xi Lin, MD, Shehla Mohammed, MD, FRCP, J. Lawrence Merritt, II, MD, Ghayda M. Mirzaa, MD, Andrew E. Timms , PhD, Joshua Scheck, B.S., Mariet Elting, MD, PhD, Abeltje M. Polstra, PhD, Lauren Schenck, MS, CGC, Maura R.Z. Ruzhnikov, MD, Annalisa Vetro, PhD, Martino Montomoli, MD, Renzo Guerrini, MD, FRCP, Daniel C. Koboldt, MS, Theresa Mihalic Mosher, MS, Matthew T. Pastore, MS, Kim L. McBride, MD MS, Jing Peng, MD, Zou Pan, MD, Marjolein Willemsen, MD, PhD, Susanne Koning, MD, Peter D. Turnpenny, MB ChB, Bert B.A. de Vries, MD, PhD, Christian Gilissen, PhD, Rolph Pfundt, PhD, Melissa Lees, MD, Stephen R. Braddock, MD, Kara C. Klemp, MS, CGC, Fleur Vansenne, MD, PhD, Marielle van Gijn, PhD, Catherine Quindipan, M.S., CGC, Matthew A. Deardorff, MD, PhD, J. Austin Hamm, MD, FACMG, Abbey M. Putnam, MS, CGC, Rebecca Baud, MS, CGC, Laurence Walsh, MD, Sally A. Lynch, MD, Julia Baptista, PhD DipRCPath, Richard E. Person, PhD, FACMG, Kristin G. Monaghan, PhD, FACMG, Amy Crunk, MS, CGC, Jennifer Keller-Ramey, PhD, FACMG, Adi Reich, PhD, Houda Zghal Elloumi, PhD, FACMG, Marielle Alders, PhD, Jennifer Kerkhof, MS, Haley McConkey, MSc, Sadegheh Haghshenas, PhD, Genomics England Research Consortium, Reza Maroofian, PhD, Bekim Sadikovic, PhD, Siddharth Banka, MBBS, PhD, Stefan T. Arold, PhD, Tahsin Stefan Barakat, PhD, MD[,#] t.barakat@erasmusmc.nl

Content

Supplementary Methods

Supplementary Case Reports

Supplementary Figures S1-S5

Supplementary Table Overview and Supplementary Table S3

Supplementary References

Supplementary Methods

Next generation sequencing analysis and recruitment of individuals

The results from Sanger sequencing confirmation can be found in Supplementary Figure S1, for those nine individuals for which this was available.

Individual 1 and 36:

Exome sequencing of DNA extracted from leukocytes was carried out for the proband and their parents using Human Core Exome (Twist Bioscience) capture followed by 2*150bp Illumina sequencing. Variant calling was performed with GATK 3.8 and variants were annotated using Alamut-Batch (v1.11). *De novo*, X-linked recessive, homozygous and compound heterozygous variants inherited *in trans* were identified for analysis in the proband using a gene-agnostic trio bioinformatics pipeline. Orthogonal validation was performed by targeted Sanger sequencing of *SETD1B* in all three family members of individual 1.

Individual 2 and individual 6

This study was approved by local institutional IRB/ethical review boards (Project ID: 07/N018, REC Ref: 07/Q0512/26), and written informed consent was obtained prior to genetic testing from the families involved. Clinical details were obtained through medical file review and clinical examination.

Genomic DNA was extracted from peripheral blood samples according to standard procedures of phenol chloroform extraction. WES on each proband was performed as described elsewhere [1] in Macrogen, Korea. Briefly, target enrichment was performed with 2 µg genomic DNA using the SureSelectXT Human All Exon Kit version 6 (Agilent Technologies, Santa Clara, CA, USA) to generate barcoded whole-exome sequencing libraries. Libraries were sequenced on the HiSeqX platform (Illumina, San Diego, CA, USA) with 50x coverage. Quality assessment of the sequence reads was performed by generating QC statistics with FastQC (http://www.bioinformatics.bbsrc.ac.uk/projects/fastqc).

Our bioinformatics filtering strategy included screening for only exonic and donor/acceptor splicing variants. In accordance with the pedigree and phenotype, priority was given to rare variants (<0.01% in public databases, including 1,000 Genomes project, NHLBI Exome Variant Server, Complete Genomics 69, and Exome Aggregation Consortium [ExAC v0.2]) that were fitting a recessive

(homozygous or compound heterozygous) or a de novo model and/or variants in genes previously linked to developmental delay, intellectual disability and other neurological disorders.

The family was collected as part of the SYNaPS Study Group collaboration funded by The Wellcome Trust and strategic award (Synaptopathies) funding (WT093205 MA and WT104033AIA). This research was conducted as part of the Queen Square Genomics group at University College London, supported by the National Institute for Health Research University College London Hospitals Biomedical Research Centre.

Individual 3:

The study was approved by the ethics committees of the Hospital District of Helsinki and Uusimaa and the Institutional review board of Columbia University, New York (IRB-AAAS3433).

For family Individual 3, DNA samples from the affected male individual and both parents underwent exome sequencing. Exomic libraries were prepared using the SureSelect Human All Exon V6 kit (60.46 Mb target region) and paired-end sequencing was performed on a HiSeq2500/4000 instrument (Illumina Inc, San Diego, CA, USA), with an average sequencing depth of on target regions of 68x. Low-quality reads were removed and the filtered reads were aligned to the human reference genome (GRCh37/Hg19) using Burrows-Wheeler Aligner-MEM (BWA)[2]. Duplicate removal, insertions/deletion (Indel)-realignment and base quality score recalibration were performed with Picard-tools and the Genome Analysis Toolkit (GATK). Single nucleotide variants (SNVs) and InDels were called by the GATK HaplotypeCaller[3]. Copy number variants (CNVs) were called in the exome data from the affected individual using CONiFER (v0.2.2)[4]. As part of the quality control, family relations and sex were confirmed using VCFtools and plink[5,6]. SNV/InDel variant annotation and filtering were performed using ANNOVAR[7] and custom scripts. Variants were filtered by first retaining exonic and splice region variants and based on variant segregation (e.g. autosomal recessive, de novo, X-linked). Next, variants with a predicted effect on protein function or pre-mRNA splicing (missense, frameshift, nonsense, start-loss, splicing, etc.) with a population specific minor allele frequency (MAF) of <0.005 (for AR) and <0.0005 (for AD) in all populations of the Genome Aggregation Database (gnomAD)[8] were retained. Last, bioinformatic prediction scores were annotated from dbnsfp35a and dbscSNV1.1 to evaluate missense and splice site variants respectively[9,10]. For CNVs, gene annotation was done using the BioMart Database[11] and variant frequency was assessed using the Database of Genomic Variants[12] and gnomAD[8] using the same frequency cut-offs as above for SNV/InDels. SNV/InDel variants were confirmed using Sanger sequencing using an ABI3130XL Genetic Analyzer. No other candidate variants than the *SETD1B* variant were identified in the analysis.

Individual 4:

Diagnostic trio whole exome sequencing was performed using the AgilentSureSelect v5 capture kit followed by sequencing on an Illumina Hiseq2500 platform (outsourced to GenomeScan, Leiden, The Netherlands). Analysis was performed in the LUMC's clinical genetic laboratory using an GATK-based pipeline and in-house developed analysis software (LOVDplus). No other candidate variants than the *SETD1B* variant were identified in the analysis.

Individuals 5, 7 and 33:

Diagnostic trio whole exome sequencing was done as previously described[13]. In short, genomic DNA was isolated from peripheral blood leukocytes of the proband and both parents, and exome-coding DNA was captured with the Agilent SureSelect Clinical Research Exome (CRE) kit (v2). Sequencing was performed on an Illumina HiSeq 4000 with 150-bp paired-end reads. Reads were aligned to hg19 using BWA (BWA-MEM v0.7.13) and variants were called using the GATK haplotype caller (v3.7[3]). Detected variants were annotated, filtered and prioritized using the Bench lab NGS v5.0.2 platform (Agilent technologies). For the Erasmus MC, use of genome-wide investigations in a diagnostic setting was IRB approved (METC-2012-387).

Individual 8 and 9:

Genomic DNA was isolated from peripheral blood leukocytes. Library preparation was using the TruSeq® DNA PCR-Free Library Prep and sequencing was on the HiSeqX machine. Reads were mapped to GRCh38 using the Isaac aligner and variants were called using the Isaac variant caller, Starling (v2.4.7, Illumina). Variants were then filtered using the Genomics England Tiering process. Individual 8 was recruited in a research study (Protocol number 11/LO/2016 Committee: NRES Committee London – Camden & Islington)

Individual 10, 13, 15, 18, 24, 30, 34 and 35:

Using genomic DNA from the proband and parents (when available), the exonic regions and flanking splice junctions of the genome were captured using the IDT xGen Exome Research Panel v1.0. Massively parallel (NextGen) sequencing was done on an Illumina system with 100bp or greater paired-end reads. Reads were aligned to human genome build GRCh37/UCSC hg19, and analyzed for sequence variants using a custom-developed analysis tool. Additional sequencing technology and variant interpretation protocol has been previously described[14]. The general assertion criteria for variant classification are publicly available on the GeneDx ClinVar submission page.

Individual 11 and 12:

Exome capture was performed with the in-house developed BCM-HGSC Core design (52 Mb; Roche NimbleGen, Madison, WI, USA), as previously described[15]. The variant calling was performed by the ATLAS2 suite[16]. Due to suspected consanguinity, the analysis focused on rare homozygous variants shared by the two brothers. Both siblings share five homozygous and two X-linked variants, including variants in three genes previously associated with human disease (*NBAS*, *NOS1*, and *SETD1B*). Bi-allelic variants in *NBAS* are associated with immune defects[17]and *NOS1* nonsense variants are associated with achalasia[18]. Both variants could hence explain part of the clinical phenotypes of these individuals but not the epilepsy and neurodevelopmental phenotypes. No other homozygous candidate variants for the epilepsy and neurodevelopmental phenotype than the *SETD1B* variant were identified in the analysis.

Individual 14, 16 and 25:

The exome was captured from peripheral blood DNA using Agilent SureSelectV6 (Agilent Technologies, Santa Clara, California) or IDT xGen Exome Research Panel (Integrated DNA Technologies, Coralville, Iowa). Subsequent paired-end sequencing was using Illumina HiSeq4000 or NovaSeq 6000 (Illumina, Santa Clara, California). Data processing, alignment (using a Burrows-Wheeler algorithm, BWA-mem) and variant calling were performed using Genome Analysis Tool Kit (GATK v4) best practices (https://software.broadinstitute.org/gatk/best-practices/) from the Broad Institute according to the reference gnome GRCh38. Variant annotation was done using ANNOVAR (http://www.openbioinformatics.org/annovar/). Variants in exonic and splicing regions were filter out with a minor allele frequency of ≤0.05 in following databases (1000G, Exome Aggregation Consortium (ExAC), the Exome Variant Server (EVS), the Genome Aggregation Database (gnomAD), and our in-house Chinese population database (CipherDB). Variants were classified as pathogenic (P), likely pathogenic (LP), variant of uncertain significance (VUS), likely benign, or benign in accordance with the guidelines of the American College of Medical Genetics and Genomics (ACMG/AMP) and recommendations by The Clinical Genome Resource (ClinGen) Sequence Variant Interpretation (SVI) Working Group (https://www.clinicalgenome.org/).

Individual 17, 27 and 29:

Saliva samples from patients and their parents were collected (Oragene DNA collection kits, DNA Genotek, Kanata, ON, Canada) and DNA extracted (QIAsymphony, Qiagen, Venlo, Netherlands); blood-derived DNA from the child was also provided by the regional genetics laboratories. DNA samples from patients and their parents were analysed at the Wellcome Trust Sanger Institute with microarray

analysis (Agilent 2x1M array CGH [Santa Clara, CA, USA] and Illumina 800K SNP genotyping [San Diego, CA, USA]) to identify copy number variants (CNVs) in the child, and exome sequencing (Agilent SureSelect 55MB Exome Plus with Illumina HiSeq) to investigate single nucleotide variants (SNVs), small insertion-deletions (indels), and CNVs in coding regions of the genome. Putative de novo sequence variants identified using DeNovoGear21 were validated with targeted Sanger sequencing. The population prevalence (minor allele frequency) of each variant in nearly 15 000 samples from diverse populations was recorded, and the effect of each genomic variant was predicted with the Ensembl Variant Effect Predictor (VEP version 2.6).

Individual 19:

Paired end reads were mapping to the human genome hg19 using BWA-MEM with default parameters, with reads being additionally processed by The Genome Analysis Toolkit (GATK) and Picard. Variants were identified using haplotype caller within GATK and Freebayes. The intersection of the two variant callers were annotated with SnpEff and loaded into a database using the GEMINI framework. Annotations included predicted functional effect (e.g., splice-site, nonsense, missense), protein position, known clinical associations (OMIM, CLINVAR), mouse phenotypes (MGI), conservation score (PhastCons, GERP), and effects protein function (PolyPhen), CADD scores, and population allele frequencies (Exome Variant Server and Exome Aggregation Consortium data). Tools within GEMINI were used to identify variants confirming to a number of disease models. We focused on variants that are rare in the population (MAF<0.01 or <0.05), are predicted to have a high impact or the gene and are de novo or transmitted in an autosomal recessive, compound heterozygote or x-linked manner[2,3]. (http://broadinstitute.github.io/picard/)

Individual 20:

Whole-exome capture and sequencing were performed using SeqCap EZ MedExome (Roche NimbleGen). The resulting libraries were sequenced on a HiSeq4000 (Illumina) according to the manufacturer's recommendations for paired-end 150 bp reads. Alignment of sequence reads to human reference genome (hg19) was done using BWAMEM 0.7.5 (bio-bwa.sourceforge.net/), and variants were called using the GATK3.3 software package (https://gatk.broadinstitute.org/hc/en-us). Filtering of variants was done using Alissa Interpret (Agilent Technologies). Variants with < 5 reads, a frequency of more than 1% in public (ESP, dbSNP, 1KG) and/or in house databases were excluded. *De novo*, homozygous or compound heterozygous variants present in exons or within +/- 6 nt in the intron were evaluated.

Individual 21:

Trio exome sequencing occurred through Invitae Laboratory (Boosted exome, trio).

Individual 22:

The study was approved by the Pediatric Ethics Committee of the Tuscany Region, in the context of the DESIRE project (Seventh Framework Programme FP7; grant agreement no. 602531). We performed trio-exome sequencing as previously reported[19] (Vetro et al, 2020). Briefly, we used the SureSelectXT Clinical Research Exome kit (Agilent Technologies, Santa Clara, CA) for library preparation and target enrichment. We sequenced the captured DNA libraries by a paired-end protocol on Illumina sequencer (NextSeq550, Illumina, San Diego, CA, USA) to obtain an average coverage of above 80x, with 97.6% of target bases covered at least 10x. We performed bioinformatics analysis by standard procedures: we aligned the sequencing reads to the GRCh37/hg19 human genome reference assembly by the BWA software package [2] and used the GATK suite for base quality score recalibration, realignment of insertion/deletions (InDels), and variant calling, according to GATK Best Practices recommendations [20]. For the annotation and filtering of exonic/splice-site single-nucleotide variants (SNVs) and coding InDels we used commercially available software (VarSeq, Golden Helix, Inc v1.4.6), focusing on non-synonimous/splice site variants with minor allele frequency (MAF) lower than 0.01 in the GnomAD database (http://gnomad.broadinstitute.org/). We further excluded population-specific variants by interrogating our internal database (WES data from over 900 patients with DEE and 200 healthy parents) and evaluated the potential functional impact of SNVs and InDels by the pre-computed genomic variants score from dbNSFP[21] which was integrated in the annotation pipeline. We also manually interrogated in-silico prediction tools [22-24], as well as evolutionary conservation scores [25,26]. For selected variants, we visually inspected the quality of reads alignment by using the Integrative Genomics Viewer[27] and then proceeded to validation by Sanger sequencing (primers and conditions are available upon request).

Individual 23:

Written informed consent was obtained for all participants in this study under a research protocol approved by the Institutional Review Board at Nationwide Children's Hospital (IRB18-00662, "Gene Discovery in Clinical Genomic Patients"). Paired-end genome sequencing libraries were constructed for DNA from the proband, mother, and father using NEBNext Ultra II FS DNA Library Prep Kit (New England BioLabs). Whole-genome sequencing was performed on an Illumina NovaSeq6000 instrument according to manufacturer protocols. Reads were mapped to the GRCh37 reference sequence and secondary data analysis was performed using Churchill[28]. The average sequence depth achieved per

sample was ~33x. Our general approach to variant annotation and prioritization has already been described[29]; for this case we prioritized rare nonsynonymous coding variants under several possible inheritance models: Dominant (de novo), recessive (homozygous or compound heterozygous), and X-linked (hemizygous). We identified two *de novo* coding variants in the proband: hg19:chr12-122261055-C-T, NM_001353345.2:c.4570C>T SETD1B:(p.Arg1524Ter) and hg19:chr1-179314193-T-C, NM_003101.6:c.1099T>C: SOAT1:(p.Phe367Leu).

Individual 26 and 28:

Diagnostic exome sequencing was done at the Departments of Human Genetics of the Radboud University Medical Center Nijmegen, The Netherlands, and performed essentially as described previously[30]. This study was approved by the institutional review board 'Commissie Mensgebonden Onderzoek Regio Arnhem-Nijmegen' under number 2011/188.

Individual 31:

Trio-exome sequencing of individual 31 occurred as previously described[30]

Individual 32:

DNA was extracted from peripheral blood using the Promega Maxwell RSC DNA Extraction Kit. The Clinical Exome Sequencing (CES) library was generated using the Agilent SureSelect Human All Exon V6 plus a custom mitochondrial genome capture kit. Captured DNA fragments were then sequenced using the Illumina Nextseq 500 or HiSeq 4000 sequencing system, with 2x100 basepair (bp) paired-end reads. Single nucleotide variants (SNVs) and small insertions and deletions (<10 bp) were detected by mapping and comparing the DNA sequences with the human reference genome (GRCh37/hg19). Variant confirmation by Sanger sequencing is performed for all insertions and deletions as well as substitutions that do not meet the laboratory's coverage and quality score thresholds.

Site directed plasmid mutagenesis and in vitro experiments

Plasmids

Human full-lengh SETD1B construct in pcDNA vector was a kind gift of Dr. David Skalnik, Indiana University[31]. Selected patient variants were introduced by site-directed mutagenesis according to the Q5® Site-Directed Mutagenesis Kit protocol (NEB). To this end the N- and C-terminal parts of SETD1B were cloned into pJet vector, variants were introduced by SDM and then ligated into the original plasmid. All created plasmids were validated by Sanger sequencing. Oligonucleotides used for SDM and sequencing are given in Supplementary Table S3 and plasmid maps are available upon request.

For bacterial protein expression the SET domain of WT or mutant SETD1B (amino acids 1727–1966) were cloned into pGEX-4T-1 GST vector (kindly provided by Dr. Mark Nellist, Erasmus MC). All plasmids were verified by Sanger sequencing.

Overexpression of Flag-SETD1B constructs in HEK cells

HEK293 LTV cells (Cell biolabs) were cultured in DMEM medium supplemented with 10% FBS at 37°C, 5% CO_2. Cultures were regularly tested for mycoplasma contamination. Cells were transfected with Flag-SETD1B plasmids at ~70% confluence using Lipofectamine3000 reagent. After 48h cells were processed for analysis by Western blot or fixed in 4% PFA for immunofluorescence staining. Confocal images were acquired with Leica Stellaris5 LIA system using LASX software.

The following antibodies were used: Rb-GAPDH (Cell Signaling, 2118S), anti-Ms-cy2 (JacksonImmunoResearch,711-225-150), anti-Rb-cy5 (JacksonImmunoResearch,711-175-152); Ms-FLAG M2 (Sigma-Aldrich, F3165); Rb-HA tag (Proteintech, 51064-2-AP); IRDye 800CW Goat anti-Rabbit (Li-cor, 926-32211); IRDye 680RD Goat anti-Mouse (Li-cor, 926-68070).

Bacterial protein expression

pGEX-SETD1B constructs were transformed into *E.coli* BL21 competent bacteria. Individual clones were picked and pre-cultures grown overnight at 37°C, 200 RPM. This culture was then used as inoculum to grow bacterial biomass until OD (A600) reached ~0.7. GST-SETD1B expression was induced with 1mM isopropyl β-D-1-thiogalactopyranoside (IPTG) for 20h at 18°C, 200RPM. Cell pellets were resuspended in the lysis buffer 50 mM Tris (pH 7.5), 300 mM NaCl, 10% glycerol, 3 mM DTT, 1 µM ZnCl2), supplemented with cOmpleteTM protease inhibitor tablets (Roche Applied Science) [32]. The cells were sonicated (2 x 30 seconds, 14 µm) and the lysates were cleared by centrifugation. The lysates were incubated at +4°C overnight on a rotation wheel with Glutathion-sepharose beads (GE Healthcare).

The beads were washed 3x with the lysis buffer and SETD1B constructs were eluted in lysis buffer supplemented with 20mM L-glutathione after 10min incubation at RT.

Thermal shift assay

Thermal shift assay was performed according to a previously published protocol[33] in a Biorad real-time thermal cycler CFX96 in transparent hard-shell 96-well plates (Biorad). The 25µl reaction contained protein of interest (final concentration 0.2ng/µl), 2.5 µl of 200x SyproOrange dye (Sigma-Aldrich), assay buffer (50mM Tris pH 7.5, 300mM NaCl). For the substrate binding test the reaction contained 100µM of S-(5′-Adenosyl)-L-methionine chloride dihydrochloride (SAM) (Sigma-Aldrich). Melt curve in the range of 20-90°C (increment of 0.2^{o}C/10sec, FRET readout) was assessed. The buffer and water controls without proteins were included in the run for background fluorescence.

Severity scoring

For each individual, a phenotype severity score (Supplementary Table S4) was calculated by adding up the main phenotypical features from Table 2, which were measured and reported in Table 1. The features taken into account were the following:

Seizure features

- Seizure: presence of seizure=1, absence=0

Development features (no=0, any affectation=1)

- Developmental delay
- Motor development
- Language development
- Intellectual disability (no affectation=0, mild=1, moderate=2, severe=3)

Behavior features (yes=1, no=0)

- Autism
- Other behavioral issues (any=1, no=0):
    - Hyperactive
    - Anxiety
    - Aggressive behavior
- Sleep disturbance

The sum was then normalized dividing it by the highest score possible for each individual. The resulting score has a range from 0 (no clinical features observed) to 1 (presentation of all examined clinical features). Scores for only development (see list above) and behavior (see list above) were also calculated by this way.

Genome-wide methylation profiles and data analysis

Genome-wide methylation profiles were obtained using the Infinium MethylationEPIC BeadChip array (Illumina)[34]. The SETD1B EpiSignature has been implemented in the clinical genome-wide DNA methylation assay, "EpiSign", and methylation profiles were analysed using the Multiclass Classification Algorithm of EpiSign v2[35].

Methylation levels calculated as the ratio of methylated signal intensity over the sum of methylated and unmethylated signal intensities, called the β-values, were converted to M-values using logit transformation in order to obtain homoscedasticity for linear regression modeling using the limma package[36]. The model matrix was constructed by these values. The estimated blood cell proportions derived by the algorithm developed by Houseman et al[37]. were added as confounding variables. Subsequently, eBayes function was operated to moderate the created p-values. In order to select the probes, we first selected 1000 probes with the highest product of methylation differences between case and control samples and the negative of the logarithm of multiple-testing corrected p-values derived from the linear modeling by Benjamini-Hochberg (BH) method. Next, a receiver's operating characteristic (ROC) analysis was performed for every probe and the pairwise Pearson's correlation coefficient between them was measured. Using the remaining ~100 probes, hierarchical clustering by Ward's method on Euclidean distance was performed using the gplots package. Multidimensional scaling (MDS) was done by scaling of the pair-wise Euclidean distances between samples.

Statistics

Statistical analysis of *in vitro* experiments used one-way ANOVA multiple comparison test or t-test as indicated in the figure legends, which also state the number of independent experiments performed. Binominal test was used for sex-specific data analysis, using all individuals from this cohort, the mother of Individual 13, and individuals from literature with reported sex[34,38-42]. Fisher's exact test was used for comparison of other clinical features between male and female patients from this cohort. The severity scores were compared by Welch Two Sample t-test. All statistical analysis was performed in Graph Pad Prism v8 software.

Supplementary Case Reports

Individual 1: c.22dup: p.(His8fs)

Individual 1 is a male, born at 38 weeks gestation. Pregnancy was complicated by gestational diabetes and intrauterine growth restriction. There were no perinatal complications. Parents and younger brother are unaffected. Early development was globally delayed. By four years challenging behaviour was prominent (sporadic tantrums, encopresis). He attended mainstream school at age five years with educational support. Cognitive assessment (WISC-IV) at age six-and-a-half years showed a full scale IQ of 57. Currently aged seven-and-a-half years he suffers from intermittent challenging behaviour, and prefers routine but does not meet criteria for autistic spectrum disorder (ASD). His motor skills are progressing e.g. tying laces, ambulating well. Examination revealed dysmorphic features including deep set eyes, short philtrum, dimpled chin, brachycephaly, cupped ear helices bilaterally with large ear lobes and marked tapering of digits. Weight accelerated from birth (2.7kg: 9th centile) to 53.3kg (>99th centile) at seven and a half years (*Figure case 1*). Neurological examination found increased tone in the left ankle. Skin examination was normal.

Seizures began at age six-and-a-half years and consisted of sudden onset, brief (up to 10 second) myoclonic absence seizures, characterised by unresponsiveness with bilateral arm/shoulder jerking and elevation, or sometimes head involvement. Seizure frequency increased over three months, appearing daily (occasionally several per hour). EEG at age 7 years showed brief generalised inter-ictal bursts of spike/polyspike and wave. Photic stimulation elicited a generalised photoparoxysmal response at 11Hz accompanied by a brief absence seizure with accompanying myoclonic activity of the upper limbs and head (*Figure case 1 F*). He was commenced on sodium valproate, which was discontinued due to worsening weight gain. He had two brief generalised tonic-clonic seizures, one of which occurred during valproate wean. Levetiracetam (24mg/kg/day) introduction led to excellent therapeutic response.

MRI brain showed bilateral sub-cortical white matter signal abnormalities (*Figure case 1 D,E*). Chromosomal microarray and Fragile X syndrome investigations were normal. Gene-agnostic trio exome sequencing analysis identified a heterozygous *de novo* novel frameshift variant in *SETD1B* [(NM_001353345.1:c.22dup p.(His8fs)], absent from the Genome Aggregation Database, predicted as likely pathogenic by introduction of a premature termination codon and production of a transcript expected to be degraded by nonsense-mediated decay.

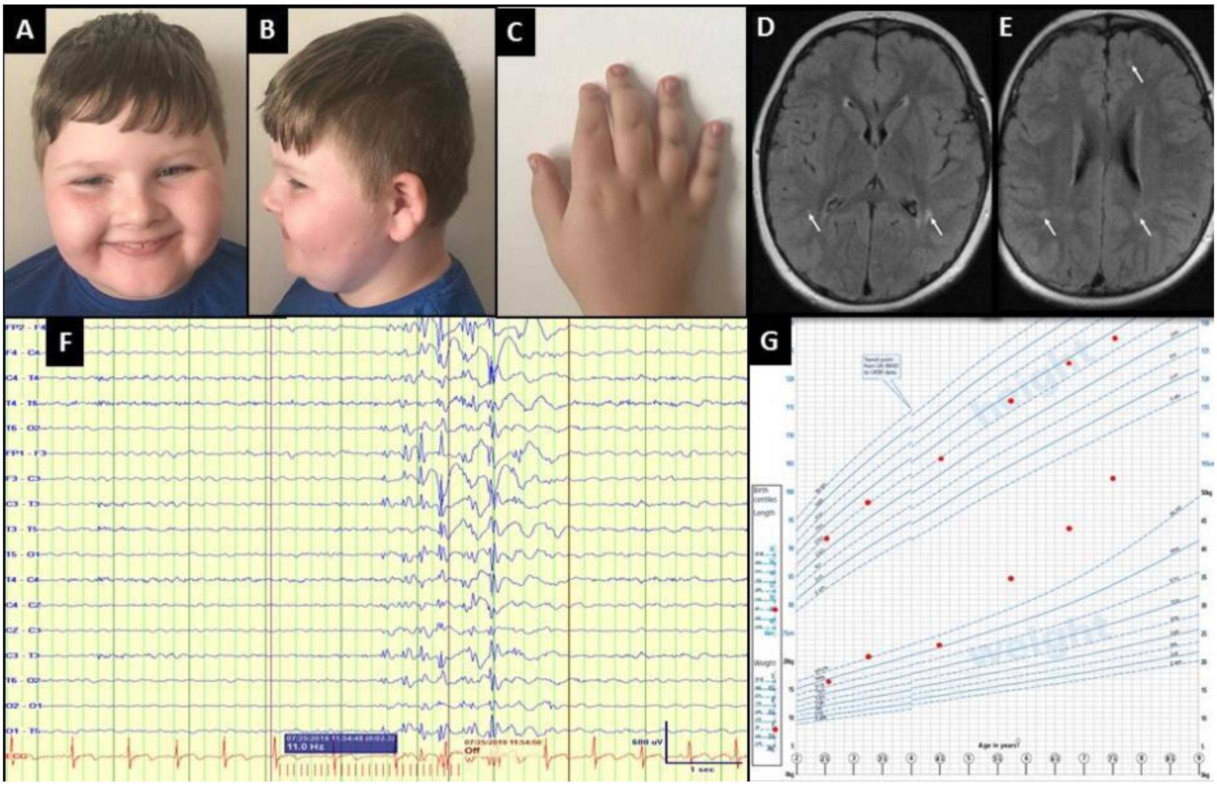

Figure case 1: A) Front profile: deep set eyes, short philtrum, dimpled chin (B) Side profile: brachycephaly, cupped ear helices and large ear lobes, (C) Tapering of digit with relatively short fifth digit. D) and E), MRI brain showing white mater subcortical hyperintensities bilaterally (noted in 3/9 patients to date). F) Ictal EEG showing brief burst of generalised 3.5-4Hz spike/polyspike and wave [accompanied by a brief myoclonic absence-jerks and brief elevation of the arms (video not available)], which coincided but was not reproduced with photic stimulation. G) Growth chart demonstrating high body mass index prior to AEDs.

Individual 2: c.22dup: p.(His8fs)

Individual 2 is a currently 10 year old female, born as the 3rd child to non-consanguineous parents from Bangladesh, who presented for genetic analysis at the age of 7 years. Her elder sibling died at the age of 6 years due to an acute abdomen, and family history was positive for a paternal uncle that was diagnose epilepsy of unknown cause which was well controlled. At the first encounter to medical investigations at 4 years of age, she showed delay in motor development, speech delay and impaired cognitive function with regression. Compared to her two siblings, she had always developed more slowly. She developed epilepsy at 4 years of age, that presented with sudden staring and head nodding, lasting for 1-2 seconds and increasing in frequency over time. An initial EEG showed transient bursts of epileptogenic discharges in sleep, but none in the awake state. On a later EEG, runs of high amplitude epileptiform discharges were noted over F3, C3, P3 and Pz. She was initially treated with

valproic acid, clorazepate, levetiracetam and risperidon, and is currently treated with valproic acid, levetiracetam and risperidon. Seizures were initially controlled under this treatment regime, but have recently re-appeared at the last investigation at 10 years of age, with an EEG showing generalized and focal discharges. Cognitive functions further regressed and she became emotional labile. At 6 years of age, a single kidney with a neurogenic bladder and a urinary bladder diverticulum were diagnosed. At the last investigation, no dysmorphisms were noted, but she has a wide spread hyperpigmented area involving the right lumbal to upper umbilical region. Her weight was 43 kg and head circumference 53 cm. She presented as playful and happy but sometimes moody. She is able to read and write limited, can talk well but was noted to have has improper emotional behavior and a low intelligence. Whole exome sequencing identified a c.22dup: p.(His8fs) variant in *SETD1B*, which was absent in both parents.

Individual 3: c.30C>A, p.(His10Gln); c.2780G>A, p.(Arg927His)

Individual 3 is a 21 years old male, the only child of the family. He was born by urgent caesarean section due placental ablation. He was diagnosed to have asphyxia, pH 6.86, from which he recovered well. His birth weight was 2930 gram, with length of 49 cm and head circumference of 35.5 cm. As a newborn he was studied due slow growth (height -4 SD, weight -1 SD). His developmental delay was noticed at 1.5 years of age. Focal epilepsy started at 2 years of age which developed to Lennox-Gastaut syndrome at 3 years of age for which Absenor and Lamictal were prescribed. He learnt to walk at 3 years of age. He speaks some words. Severe intellectual disability was diagnosed at 4 years of age. His adult height is 158 cm. He has difficulties in walking due hypotonia. His balance is weak and he has a tendency to fall and hurt himself. He has also intention tremor. At 11 years of age aseptic coxitis was detected. His facial features include a narrow skull, narrow and high palate and small, low set ears. His fingers are short and he has a sandal gap between 1. and 2. toes. He had enuresis until 18 years of age. His kidney ultrasound is normal. In EEG interictal spikes are characteristic (Figures Case 3). When awake occasional spikes from both hemispheres, especially from centroparietal areas are detected, whereas during sleep abundant multifocal spikes, especially from centroparietal areas (right side often more affected) are found. In EEG background no reaction to eye opening or sleep phenomena were found (Figure Case 3). Findings were similar in repeated recordings over the years. Brain MRI and ENMG were normal. Screening of urine amino-acids, oligosaccharides and glycosaminoglycans was normal. A HumanCytoSNP-12 (v2.1) (Illumina) was normal. Subsequent trio exome sequencing identified compound heterozygote variants (p.His10Gln and p.Arg927His) in the *SETD1B* gene. The parents are heterozygous for these variants and healthy.

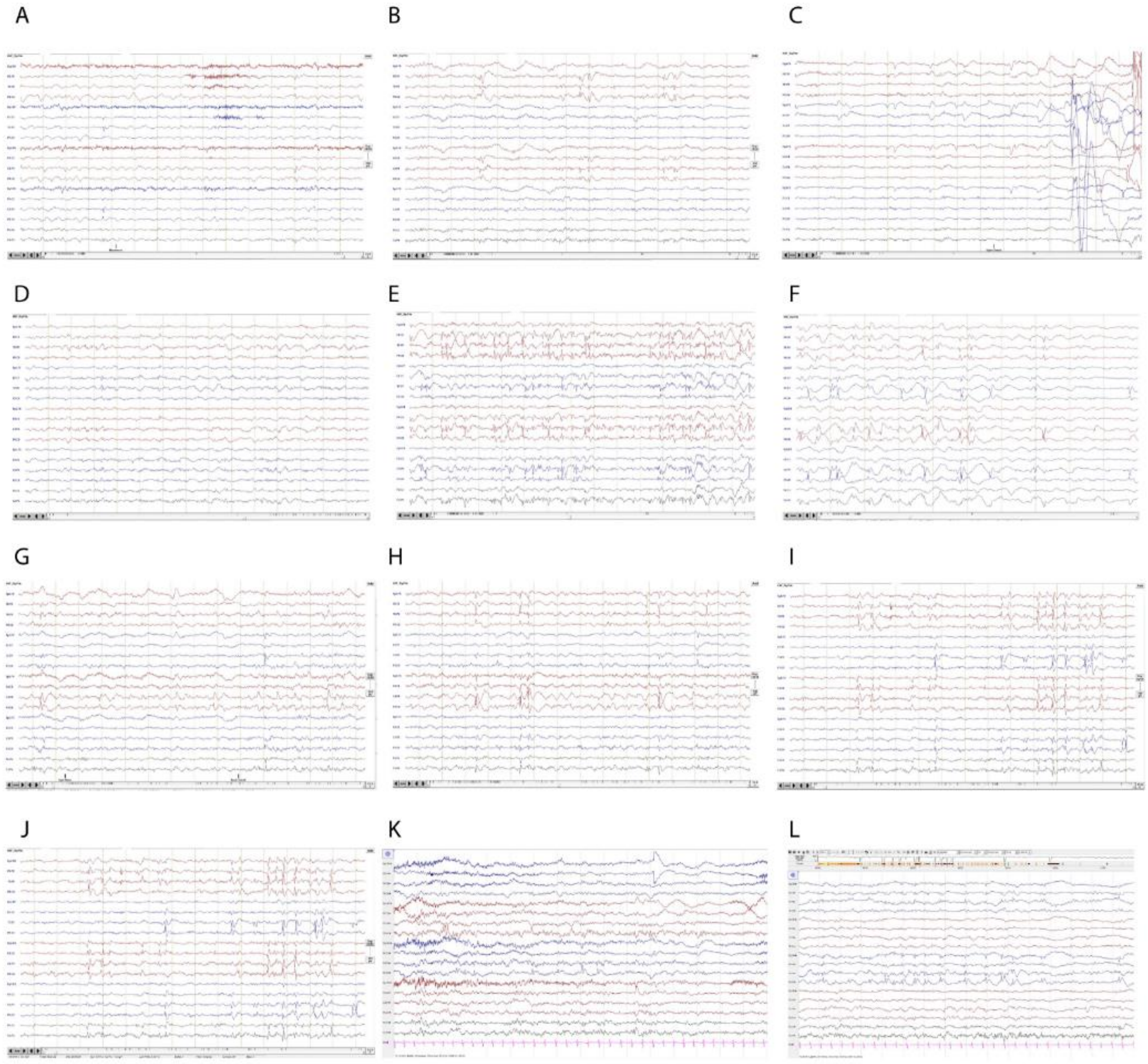

<u>Figure Case 3</u>: Repeated EEG recordings over the years, at awake and sleeping state, showing mainly multifocal spikes at the centroparietal areas. A) and B) age of 6 years, awake, showing occasional multifocal spikes especially at the centroparietal areas. C) age of 7 years, showing continuous EEG background, without posterior rhythms during eye closure. D) age of 6 years, possible during sleep, showing spikes at the centrotemporal areas. E) and F) during sleep at the age of 6 years, showing abundant spikes from the centroparietal areas. G) Continuous EEG at the age of 8 years, awake; H) age 8 years, possible sleeping. I) and J) 9 years of age during sleep. K) age of 10 years, awake and L) age of 10 years, during sleep, showing right parieto frontal spikes (P4-F4), and left sides multifocal spikes (P3, C3, T5).

Individual 4: c.282G>C, p.(Glu94Asp); c.3982C>T, p.(Pro1328Ser)

After an uneventful pregnancy and home delivery, a boy was born at gestational age of 41+1 weeks with a birth weight of 4.5 kg. On day 1 after birth he experienced episodes of apnea with desaturation and tonic seizures. A cerebral ultrasound and MRI were performed, showing cystic encephalomalacia at the right side of the brain with bilateral ventriculomegaly and displacement of the brainstem. Fenestration of the cyst occurred and an Ommaya shunt was inserted. Seizures were treated with phenobarbitone for 2 months. At the age of 11 months focal motor seizures reoccurred, and antiepileptic drugs were restarted (valproic acid and clobazam). In the year thereafter, valproic acid was switched to oxcarbazepine with good effect. Because of exotropia, he had strabismus surgery at the age of 6 years. He developed some form of speech (copying words and singing short lines) from the age of 2 years, but lost this ability once he started walking. His motor development was severely delayed (ambulation at 4 years). Currently, at the age of 11, he produces sounds but no words. Furthermore, he has pronounced joint hypermobility and severe constipation, for which a high dose macrogol is required. He has autistiform behavior but does not meet diagnostic criteria. Behavioral and sleeping problems seem to increase throughout the winter period.

Individual 13: c.1234del, p.(Glu412fs)

Individual 13 is a 5 year old male with a dual diagnoses of *PTEN*-related disorder and *SETD1B*-related disorder. He is also a heterozygous for sickle cell trait. His associated features include macrocephaly, rapid growth, developmental delay with regression, epilepsy, and severe autism.

Whole exome sequencing, which revealed a reclassification of the *PTEN* variant from variant of uncertain significance to likely pathogenic, as well as a maternally inherited pathogenic variant in *SETD1B* (c.1234del, p.(Glu412fs)). It also confirmed his previously known sickle cell trait. The mother is similarly affected with borderline intellectual functioning (IQ72), seizures, and autism spectrum disorder; she is immature for age and has prominent facial features and some slight facial asymmetry, and shares her son's very straight eyebrows. She is not unaffected, though clearly more high functioning than her affected son. The maternal grandmother did not carry the *SETD1B* variant, but reports a history of learning disability and childhood seizures in herself. She currently functions as the guardian of daughter and grandson.

Of note, the patient is also status post normal chromosome microarray, fragile X testing, MPS screening, inborn errors of metabolism screening, and brain MRI. Most recent thyroid ultrasound was normal.

Individual 14: c.1285C>T, p.(Arg429Trp)

Individual 14 is a currently 2 years and 3 months old female, born to non-consanguineous Chinese parents, at 40+2 weeks of gestation, with a birth weight of 2.7 kg and a length of 50 cm. Developmental milestones were achieved normally, and no dysmorphic features were noticed. A first seizure occurred at the age of 1 year and 11 months old. Her symptoms started with vomiting and diarrhea, followed by her eyes turned upwards, lips and face turned to pale, generalized convulsions lasted for one minute. Second seizure occurred again recently, with similar symptoms: eyes turn up, left upper limb flexed and raised, chewing, salivation, cyanosis of the lips, and then the whole body twitches, which lasted around 90 seconds. She was treated with phenobarbital orally, and is seizure free so far. Brain imaging was unremarkable.

Individual 15: c.1634C>G, p.(Pro545Arg)

Individual 15 is a 19 year-old male presented to the Genetics Clinic at 18 years of age, referred by his neurologist for consultation due to his diagnoses of autism spectrum disorder, attention deficit hyperactivity disorder, seizure disorder, and mild intellectual disability. Prior to his Genetics visit, his neurologist ordered a karyotype that revealed an apparently balanced reciprocal translocation between chromosomes 12 and 19 - 46,XY,t(12;19)(p11.2;p13.1). He had a normal microarray. The patient was adopted at 6 months of age from Russia and there is no prenatal history or early developmental history known prior to 6 months. He had onset of seizures at age of 6 years and currently has absence seizures occurring around once a month (well-managed on Keppra and Depakote). He is generally non-dysmorphic on physical exam. Fragile X testing and an epilepsy panel were ordered following his Genetics visit and were both negative. At that time, whole exome sequencing was ordered and a variant of uncertain significance (c.1634C>G, p.(Pro545Arg) was identified in the *SETD1B* gene.

Individual 16: c.2092C>T, p.(Pro698Ser)

Individual 16 is a currently 1 year old female, born as the first child to non-consanguineous Chinese parents at 38+1 weeks of gestation, with a birth weight of 2.26 kg and a good start (APGAR 10/10/10). Delivery was by cesarean section due to mother's preeclampsia. After birth, she was hospitalized for treatment because of neonatal pneumonia, hyperglycemia, conjunctivitis, low birth weight and patent arterial duct. One day before she was admitted to hospital at the age of 3 months and 12 days, she had high fever (39.6 C) with unknown cause. Parents tried to physically lower her body temperature by rubbing with alcohol and treated her with antibiotics, and her body temperature decreased to normal gradually. 12 hours before admission, she started with sighing breath, lasted for 5-10 minutes,

followed by seizures unconsciously with right upper limb convulsion, left upper limb and both lower limbs tonic-clonic seizures, while head moving leftwards, both eyes gazing leftwards, with a pale face, purple lips, and tightly closed mouth. Seizure lasted about 10 minutes, and her body temperature was 37.9 C at that time. She was admitted to the hospital unconsciously. Lung CT showed signs of a bi-lobular pneumonia and blood work suggested mild anemia and septicemia. She can raise her head steadily by 3 months, is able to smile, but not very active, not very good at following light or moving objects. Her physical examination showed hypertonia, with high deep tendon reflexes (knee and Achilles) and a possibly positive Babinski signs. No dysmorphic features were observed. Brain MRI showed bilateral abnormal signals at temporal, occipital lobes. Current development at 1 year old seems normal. Whole exome sequencing identified a *de novo* c.2092C>T, p.(Pro698Ser) variant in *SETD1B*.

Individual 21: c.3985C>T, p.(Arg1329)*

Individual 21 is a 4-year-old female with predominantly myoclonic epilepsy and developmental delays. Her early development involved slight delays in walking and language, and typical fine motor skills. Speech and language assessments have shown slightly below average expressive and receptive language skills. She receives occupational and speech therapy. She has not demonstrated any developmental regression. The subject was initially diagnosed with absence seizures with eyelid myoclonia at 2 years old, which evolved into very frequent treatment refractory epilepsy. She typically has around 70-100 myoclonic seizures per day; these frequent myoclonic seizures have been demonstrated on EEG. Prior AEDs trialed include Lamicatal, Diamox, which seemed to possibly increase seizures, and Onfi. She is currently treated with Keppra and Epidiolex, and is on a modified Atkins diet. She seems to have more seizures in the morning or evening and during sedentary activities, like watching tv. Brain MRI at 4 years old revealed right choroid fissure cyst, and subtle features of increased FLAIR signal that may represent slightly delayed myelination and questionable small heterotopic gray matter. The child has not yet had a full dysmorphology exam in clinic. Over telehealth appointment she was noted to have light blond hair, normally shared ears, normal palmar and foot markings, typical gait, and typical facies. Her prior genetic work-up included an epilepsy panel with a variant of unknown significance in the gene CHRNB2, which was considered non-diagnostic. She was also identified to be heterozygous for a Fragile X premutation (57 and 29 CGG repeats). Trio exome sequencing identified a *de novo c.3985C>T, p.(Arg1329*)* nonsense variant in *SETD1B*.

Individual 22: c.4271G>A, p.(Arg1424Gln)

Individual 22 is a 3 years and 6 months old boy, first child of healthy non-consanguineous Italian parents. Pregnancy was unremarkable and delivery was by induced labor at 39+6 weeks. Birth weight (3615 gr, +0.11 SD), length (51 cm, +0.32 SD) and head circumference (35 cm, -0.40 SD) were within normal range. Global hypotonia and developmental delay were noticed since age of 4 months. The child achieved head control at 1 year and 3 months and independent walking at 3 years. Neuropsychological evaluation at 2 years and 4 months was consistent with moderate developmental delay (Bayley III scale: Cognitive, 55; Language, 49; Motor, 42). Two brain MRI scans, performed at 1 year and 3 years, were normal. At age 3 years, the boy was still non-verbal and manifested brief episodes of unresponsiveness with eyelid myoclonia, accompanied by generalized spike-wave discharges. Treatment with valproate was rapidly beneficial.

Plasma metabolic workup and SNP-array analysis were normal. Whole exome sequencing uncovered the *de novo* c.4271G>A [p.(Arg1424Gln)] *SETD1B* variant of (accession nr. NM_001353345.1), which was classified as likely pathogenic according to ACMG/AMP (PS2, PM2, PP3). The variant was not observed in the allele frequency database GnomAD (v2.1) and was predicted to be damaging by multiple *in silico* bioinformatics tools (SIFT, Polyphen2, MutationTaster).

Individual 23: c.4570C>T, p.(Arg1524)*

Individual 23 is a currently a 5-year-old Caucasian male born at 38 weeks gestation. The pregnancy was complicated by maternal factor V Leiden deficiency, for which mother took baby aspirin during the pregnancy. Other maternal medications included progesterone injections until 12 weeks. Intrauterine growth retardation was noticed, and labor was induced at 38 weeks due to this. Patient weighed 5 pounds, 7 ounces at birth (2.466 kg, 4.19%, Z=-1.73), and was 17.5 inches long (44.5 cm, 2.0%, Z=-2.05). Global developmental delays were noted early on, with the patient not sitting up on his own until 9 months, or crawling until a year of age. Patient began receiving therapies (physical and occupational) at 9 months of age. More severe delays in speech were noted as patient got older, and speech therapy was engaged. Patient began speaking around 2 years of age, and was still using only single-words at 3 years of age. He was diagnosed with apraxia, global developmental delays, and ADHD. At age 4, he had a full developmental and psychological assessment confirming his diagnoses of global developmental delay and ADHD. He had a full-scale IQ of 63 on the Leiter-3 scale at that time. Medically, he has been generally healthy, but did have conductive hearing loss due to fluid in his ears, for which he received ear tubes, and enlarged tonsils, for which he had a tonsillectomy. His current growth parameters at

age 5 years, 10 months are 53 pounds, 5.6 ounces (24.3 kg, 86.86%, Z=1.12) and 47.72 inches tall (121 cm, 90.35%, Z=1.30).

The patient, along with his sister and parents, were enrolled in a research study for whole genome sequencing, and the c.4570C>T, p.(Arg1524*) variant in *SETD1B* was found to be *de novo* in the male patient. Parents are both healthy with normal cognition, and there is no additional family history of intellectual disability or birth defects. Consanguinity was denied.

Individual 24: c.4996C>T, p.(Gln1666)*

Individual 24 is a 14-year-old male with generalized epilepsy and mild intellectual disability. His early development was notable for expressive language delay; gross motor and fine motor skills were attained towards the end of the normal range. Notable challenges in academic skills became apparent with initiation of formal schooling, primarily affecting writing, comprehension, and memory recall. There has been no history of developmental regression. At 6 years of age, it was recommended that he undergo EEG, which demonstrated brief absence seizures occurring every 30-90 seconds. Given their subtle semiology, seizure onset is not known but is thought to be prior to 6 years. The child has since trialed multiple antiepileptic drugs (AEDs) and had a vagus nerve stimulator (VNS) placed at 11 years of age. He continues to have daily clusters of seizures upon awakening with his current AED regimen of cannabidiol, clobazam, and ethosuximide. Known seizure triggers include dehydration, sleep deprivation, and emesis. The subject continues to have difficulty with schoolwork and his most recent neuropsychiatric evaluation revealed a full scale IQ consistent with mild intellectual disability. His neurologic exam was also significant for hypotonia (appendicular > axial) and intermittent tremor. Several minor anomalies were noted on dysmorphology exam, including: low anterior hairline, bilateral posterior helical ear pits, mild synophrys with broad eyebrows, upslanting palpebral fissures, an upturned nose with broad nasal tip, and wide mouth. His prior genetic work-up included a SNP chromosomal microarray and a large epilepsy gene panel. Trio whole exome sequencing identified a *de novo* c.4996C>T, p.(Gln1666*) variant in *SETD1B*.

Individual 27: c.5374C>T, p.(Arg1792Trp)

Individual 27 is a currently 13 year old female, born at full term weighting 3033 gram, to non-consanguineous parents. During the first 4-6 post-conception weeks of pregnancy mother took recreational drugs (cocaine, cannabis, ecstasy) and alcohol but stopped immediately on learning she was pregnant. Parturition was prolonged and she was a vaginal birth with occiput-posterior. Placenta accreta was noted but there were no neonatal problems and she was breast-fed for 12 months. She was a placid infant who fed well, sat independently at 9 months, walked independently at 20 months,

and was late developing babble and speech. She was noted to be hypotonic in infancy and through childhood, with longstanding coordination difficulties and mild ataxia. Her seizure disorder probably started around 6 months of age with absences and was formally diagnosed at 18 months. While presenting mainly as absences her generalized seizure disorder (as suggested by her EEG) can include tonic-clonic episodes. The absences can be very frequent and have been difficult to control, with no significant improvement on either sodium valproate, lamotrigine, or levetiracetam. Some improvement has been manifest on ethosuximide and topiramate. She has mild intellectual disability and speech delay, and communication and social difficulties thought to be consistent with autistic spectrum disorder; however, on formal assessment she was considered not to have autism. She manifests hyperacusis, and has outbursts of hyperactivity as well as aggression. She is medicated for constipation and has episodes of fecal soiling. She has always fed well and been well grown, with height and weight approximately 95th centile throughout childhood. Head circumference was 51.0 cm at age 6 years and 2 months. Parental heights differ markedly: mother ~138 cm (OFC 55.8 cm); father ~188cm (OFC 55.7cm). She is not obviously dysmorphic but has mildly anteverted nares and slightly short fingernails and terminal phalanges. She was noted to have mild 5th finger brachyclinodactyly when younger, and at age of 6 years her palpebral fissure length was on the 5th centile. Through the Deciphering Developmental Disorders project she was found to have the *de novo* heterozygous missense *SETD1B* variant: c.5374C>T, p.(Arg1792Trp).

Individual 30: c.5702C>T, p.(Ala1901Val)

Individual 30 was a male born at 40 2/7 weeks gestation to a 31-year-old female. A fertility herbal blend was ingested by the mother and the father prior to conception. Medications during pregnancy included prenatal vitamins, Valtrex, Glyburide, and Zofran. Hypoplastic left heart syndrome was identified at 22 weeks gestation by fetal echocardiogram. Pregnancy was also complicated by maternal gestational diabetes.

He was born by induced vaginal delivery with a birth weight of 3340 gram, length of 51.5 cm, and head circumference of 33.5 cm. On a postnatal echocardiogram, the diagnosis of hypoplastic left heart syndrome was confirmed, in addition to identification of a moderate-sized atrial septal defect, severe hypoplasia of the ascending aorta (4 mm), moderate hypoplasia of the aortic arch, and patent ductus arteriosus. On day 6 of life, he underwent a hybrid procedure with bilateral pulmonary artery banding and placement of a single ductal stent. At 3.5-months-old, he underwent a multi-procedure surgery that included tricuspid valve repair, removal of ductal stent, patch enlargement, atrial septectomy, Damus-Kaye-Stansel connection, right-sided bidirectional Glenn shunt, and balloon dilatation of the left pulmonary artery. Completion Fontan procedure was performed at 2-years-old. Additional cardiac

catheterizations were performed at 1-month-old, 3-months-old, 2-years-old, and 3-years-old. He was hospitalized once after the completion Fontan procedure due to an upper respiratory infection. His cardiovascular status has remained stable.

He experienced developmental delay. He rolled over at 6 months, sat by himself and crawled at 12 months, walked unassisted at 18.5 months, and spoke his first word at 18 months. Verbal regression characterized by the loss of approximately 30 words occurred around 24-months-old. He was diagnosed with autism spectrum disorder by a developmental pediatrician at 3-years-old. Brain magnetic resonance imaging identified mild lateral and third ventricular enlargement with mild periventricular white matter thinning and minimal periventricular white matter FLAIR hyper-intensity suggesting a mild form of periventricular leucomalacia, in addition to several foci of susceptibility artifact scattered in both cerebral hemispheres.

Family history was significant for early-onset breast cancer. His mother was diagnosed with unilateral breast cancer at 28-years-old. Family history was negative for additional cases of congenital heart defect, developmental delay, and autism spectrum disorder. There was no reported consanguinity or Ashkenazi Jewish ancestry.

He had a negative karyotype, microarray, Fragile X testing, and FISH for 22q11.2 deletion. Clinical exome sequencing reported a de novo novel missense variant, c.5702C>T: p.Ala1901Val, in *SETD1B*.

Individual 32: c.5820_5826del, p.(Tyr1941fs)

Individual 32 is a currently 15-year-old boy recently diagnosed with a *de novo SETD1B* c.5820_5826delCTATGAC, p.Tyr1941fs variant. He presented at 1-1/2 years of age with medically intractable Lennox-Gastaut syndrome. He was noted to be globally developmental delayed since infancy and is affected with intellectual impairment and autistic features. Currently he is becoming less verbal and there is a concern for a regression of expressive language skills. He has fuller cheeks, tapered fingers, thoracolumbar scoliosis and pes planus.

Individual 34: c.5842G>A, p.(Glu1948Lys)

Individual 34 presented to the hospital as 18 days old male with apneic episodes associated with micrognathia, laryngomalacia, and symptoms of gastoesophageal reflux disease. Laryngomalacia was corrected with supraglottoplasty at 3 months old. By 23 months old, he demonstrated motor and language delay and had been diagnosed with sensory processing disorder. MRI of the brain performed due to history of suspected vestibular dysfunction demonstrated normal results, and there were no

abnormalities on an EEG that was conducted during a sleep study. At 30 months old the patient had been formally diagnosed with autism spectrum disorder and did not have history of seizure activity.

Individual 35: c.5842G>A, p.(Glu1948Lys)

Individual 35 has a history of early developmental delay, medically refractory absence epilepsy with a myoclonic component, and autism spectrum disorder. He was delivered at term with a birth weight of 7 pounds 11 ounces. He experienced early GE-reflux and underwent a frenectomy as an infant. He had early developmental issues and did not walk until starting early intervention therapies at 14 months of age. He also had a significant language delay. His parents began to notice brief staring spells sometimes with a larger jerk in his infancy. This was subsequently diagnosed as epilepsy around the age of 3 years. He was found on EEG to have generalized polyspike spike-wave discharges. Since then he has had medically refractory absence epilepsy with a myoclonic component but also has had generalized tonic-clonic seizures. He was thought initially not to have autism, but as he got older his processing issues and perseverative behaviors became more evident and he was diagnosed with autism spectrum disorder at the age of 4. He has never had regression.

Supplementary Figures

Supplementary Figure S1: Sanger sequences of selected *SETD1B* variants. Shown are the chromatograms of affected individuals and family members, as indicated. For individual 22, also the IGV view of the trio is shown.

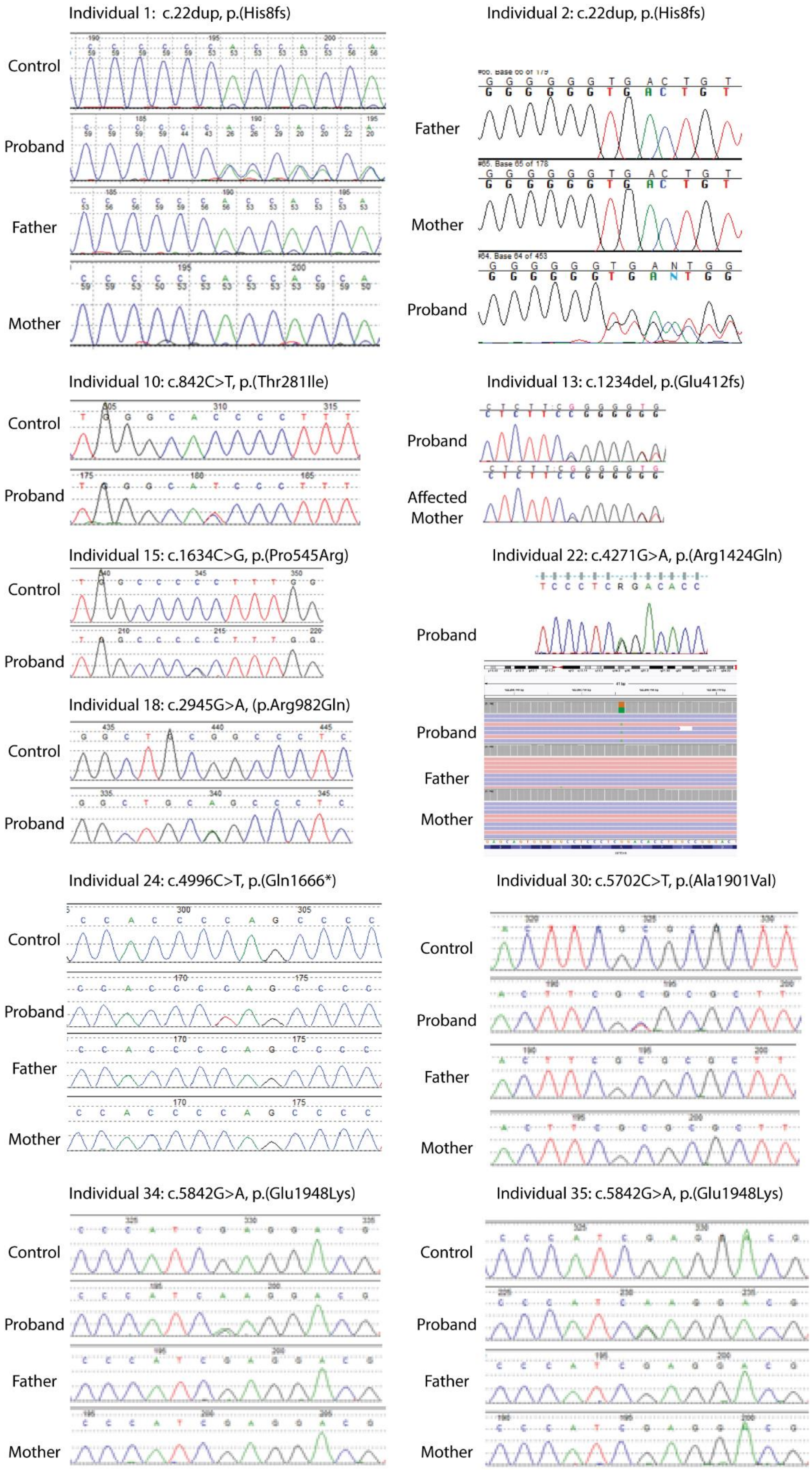
Individual 1: c.22dup, p.(His8fs)
Control
Proband
Father
Mother
Individual 2: c.22dup, p.(His8fs)
Father
Mother
Proband
Individual 10: c.842C>T, p.(Thr281Ile)
Control
Proband
Individual 13: c.1234del, p.(Glu412fs)
Proband
Affected Mother
Individual 15: c.1634C>G, p.(Pro545Arg)
Control
Proband
Individual 22: c.4271G>A, p.(Arg1424Gln)
Proband
Proband
Father
Mother
Individual 18: c.2945G>A, (p.Arg982Gln)
Control
Proband
Individual 24: c.4996C>T, p.(Gln1666*)
Control
Proband
Father
Mother
Individual 30: c.5702C>T, p.(Ala1901Val)
Control
Proband
Father
Mother
Individual 34: c.5842G>A, p.(Glu1948Lys)
Control
Proband
Father
Mother
Individual 35: c.5842G>A, p.(Glu1948Lys)
Control
Proband
Father
Mother

Supplementary Figure S2: Brain MRI imaging of selected individuals with SETD1B variants.

Shown are representative MRI brain images (T1 or T2 weighted) in different planes, for individuals 1, 4, 10, 13, 16, 18, 22 and 24, showing non-specific minor subcortical white matter hyperintensities (individual 1), a cystic encephalomalacia in right hemisphere with ventriculomegaly and the need for a shunt (individual 4), reduced white matter and thin corpus callosum (individual 10), bilateral abnormal signals at temporal and occipital lobes (individual 16), or unremarkable findings (individual 13, 18, 22, 24). P: pathogenic variant, LP: likely pathogenic variant, V: variant of uncertain significance.

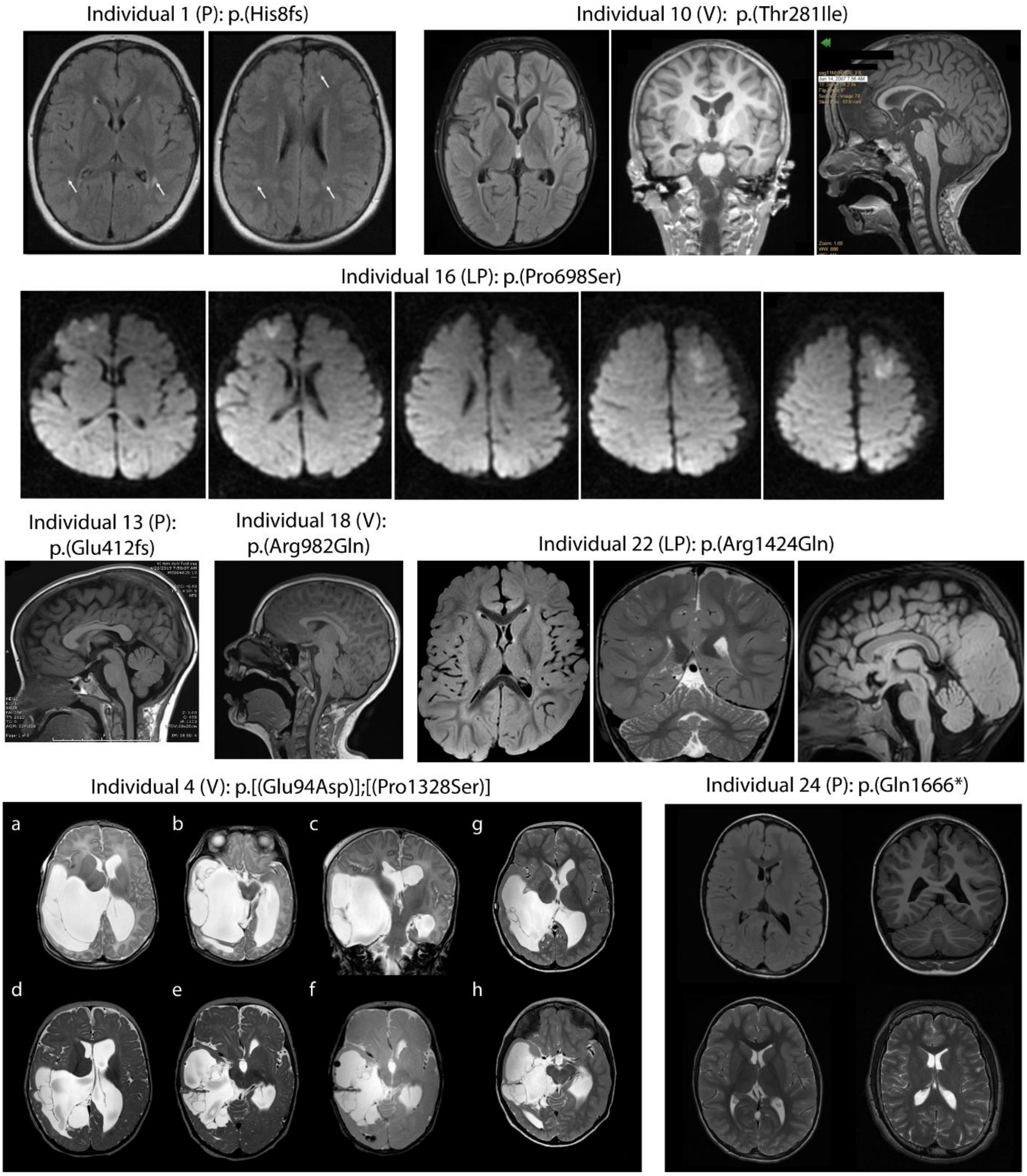

Supplementary Figure S3: Photographs of hands and feet of the indicated individuals. P: pathogenic variant, LP: likely pathogenic variant, V: variant of uncertain significance.

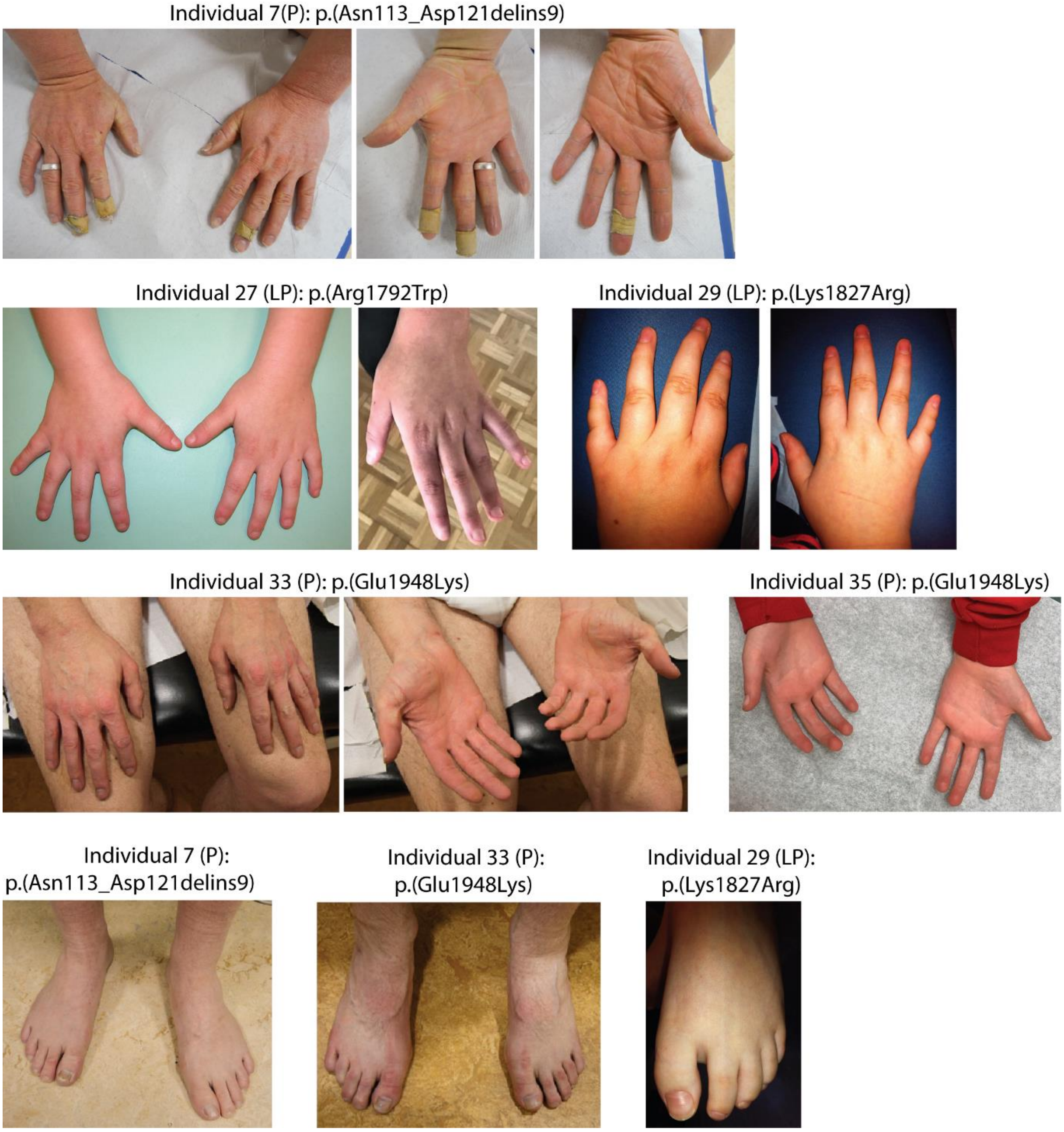

Supplementary Figure S4: **Functional evaluation of SETD1B variants**

(A) Full western blot (triplicate) of overexpression of wild type and variant SETD1B protein in HEK293 cells 48h post-transfection assessed by Western blot. CC- cell control, lysate of mock transfected HEK293 cells. (B) Representative western blot of wild type and variant SETD1B protein in triplicates. (C) Western blot of GST-SET domain of SETD1B expressed in *E.coli* BL21, soluble fraction of protein extracts. (D) Representative melting curves of thermal shift analysis of GST-SET domain proteins. (F) Methylation variant pathogenicity scores for methylation profiles.

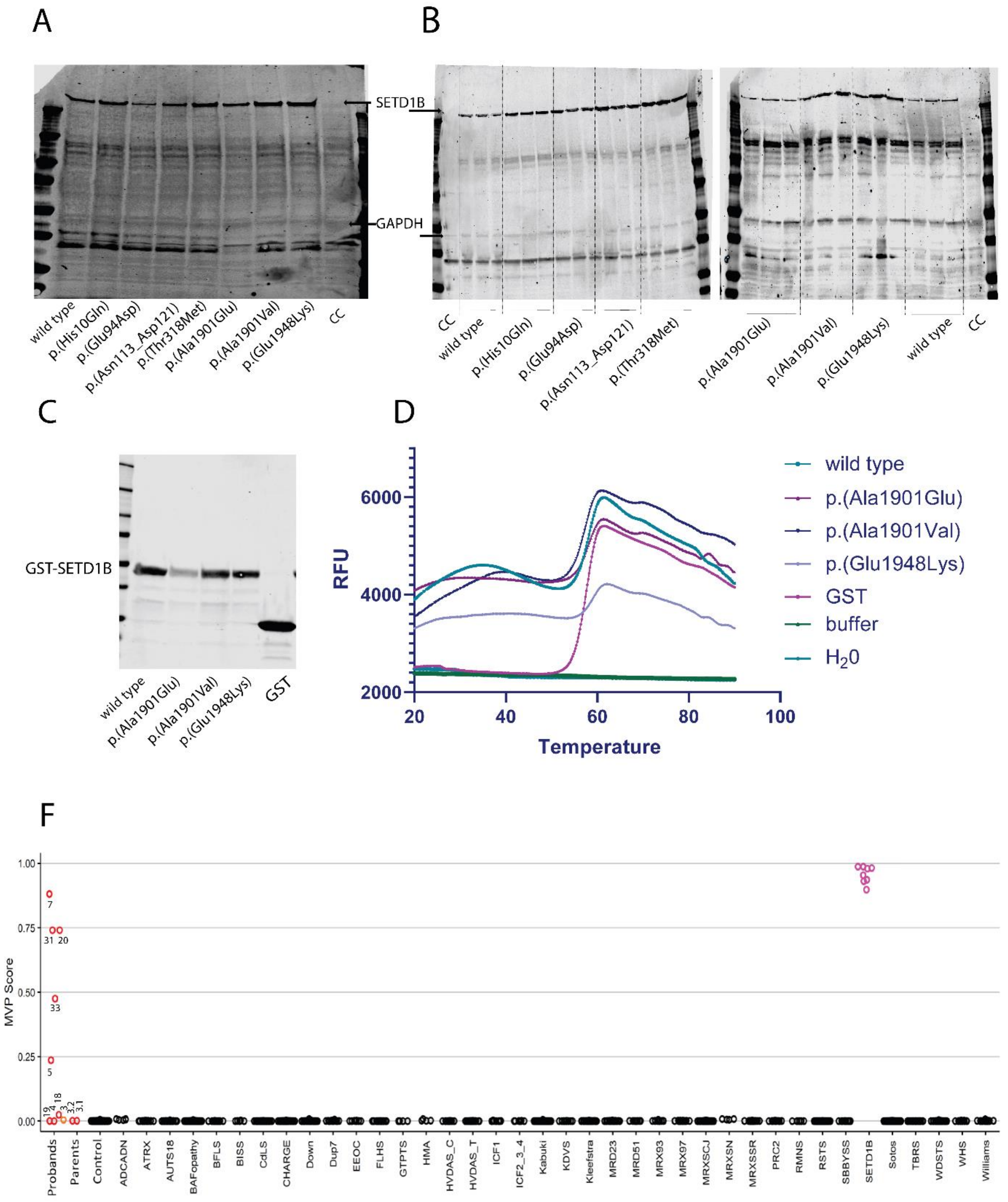

A
SETD1B
GAPDH
wild type
p.(His10Gln)
p.(Glu94Asp)
p.(Asn113_Asp121)
p.(Thr318Met)
p.(Ala1901Glu)
p.(Ala1901Val)
p.(Glu1948Lys)
CC
B
SETD1B
GAPDH
CC
wild type
p.(His10Gln)
p.(Glu94Asp)
p.(Asn113_Asp121)
p.(Thr318Met)
p.(Ala1901Glu)
p.(Ala1901Val)
p.(Glu1948Lys)
wild type
CC
C
GST-SETD1B
wild type
p.(Ala1901Glu)
p.(Ala1901Val)
p.(Glu1948Lys)
GST
D
RFU
6000
4000
2000
20
40
60
80
100
Temperature
wild type
p.(Ala1901Glu)
p.(Ala1901Val)
p.(Glu1948Lys)
GST
buffer
H20
F
MVP Score
1.00
0.75
0.50
0.25
0.00
7
31 20
33
5
19
4
18
3
3.2
3.1
Probands
Parents
Control
ADCADN
ATRX
AUTS18
BAFopathy
BFLS
BISS
CdLS
CHARGE
Down
Dup7
EEOC
FLHS
GTPTS
HMA
HVDAS_C
HVDAS_T
ICF1
ICF2_3_4
Kabuki
KDVS
Kleefstra
MRD23
MRD51
MRX93
MRX97
MRXSCJ
MRXSN
MRXSSR
PRC2
RMNS
RSTS
SBBYSS
SETD1B
Sotos
TBRS
WDSTS
WHS
Williams

Supplementary Figure S5: **Average severity score in individuals with heterozygous variants.**

Severity scores were calculated for each individual, and the average for each category is shown only for heterozygous variants. A) Severity score for categories for variants based on predicted effect on protein or stability (left plot, I=Catalytic site and/or substrate binding, II=Stability of SETD1B / complex formation / other protein-protein interactions, III=Truncation, IV=No apparent effect), functional status of the gene product (center plot, loss-of-function (LOF), diminished function (DF), or uncertain) or affected region (right plot, 1=All domains affected; 2=Middle, N-SET and SET domains affected; 3=N-SET and SET domains affected; 4=Middle region affected; 5=RRM domain affected). Numbers in parenthesis present the numbers of variants for each category. No statistically significant differences were observed (Welch Two Sample t-test p>0.05). B) Heterozygous male (n=20) and heterozygous female (n=12) comparison for clinical features (left), only features related to development (center) or features related to behavior (right). The male and female groups are statistically different in the overall score (p=0.025) and in the behavior score (p=0.006) but not in the development score (p=0.5) (Welch Two Sample t-test).

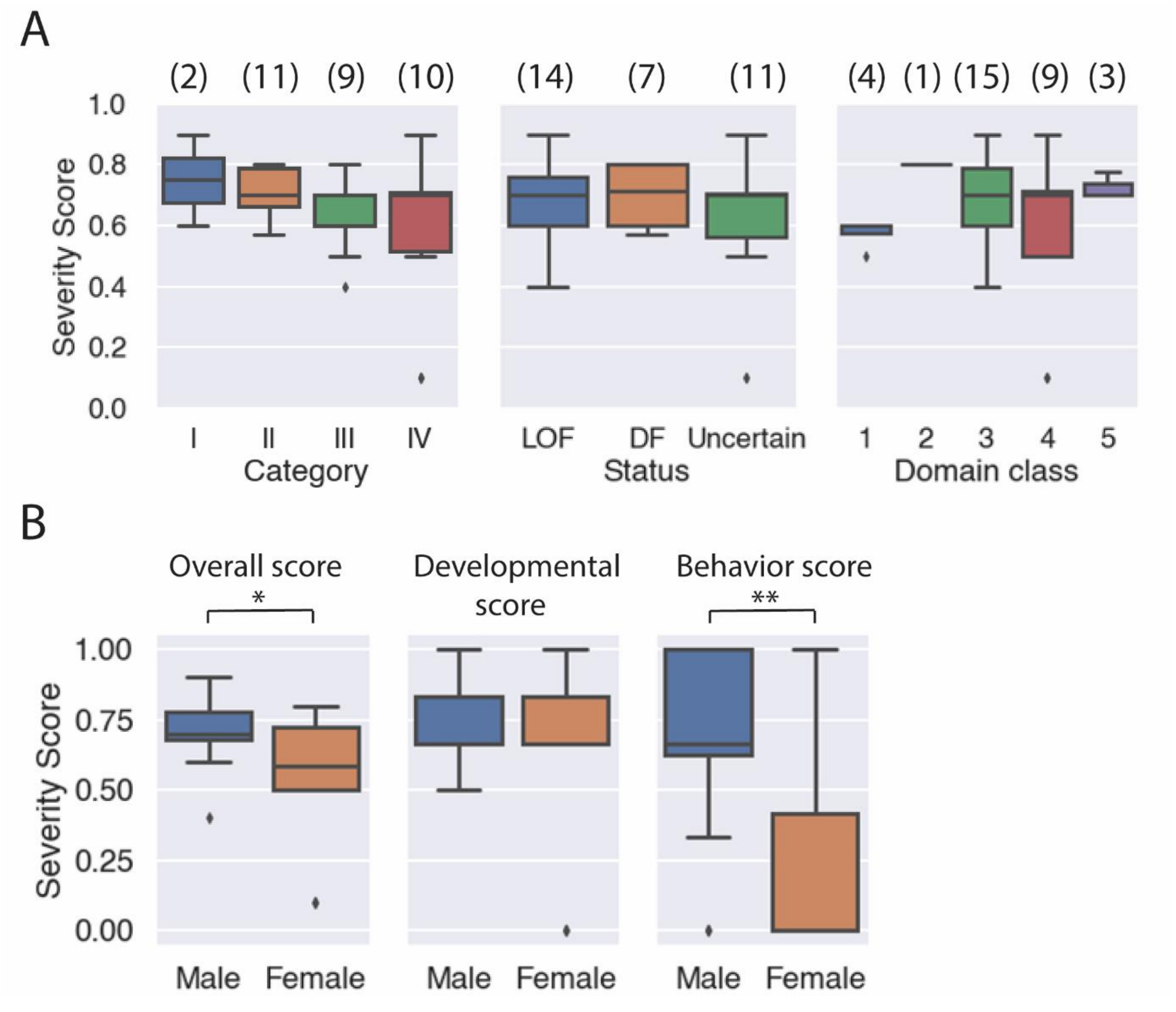

Supplementary Table Overview and Supplementary Table S3

Supplementary Table S1: Classification of all SETD1B variant (cohort and literature).

(provided as separate file) <<Supplementary Table S1_ACMG_AMP classification.xlsx>>

Supplementary Table S2: Anthropomorphic measurements.

(provided as separate file) <<Supplementary Table S2_Anthropomorphic.xlsx>>

Supplementary Table S3: Oligonucleotides for site directed mutagenesis.

**Oligonucleotide name**	**sequence**
B996 SETD1B_His10Gln_F	CCCACCACCAaCACCAGCAGC
B997 SETD1B_His10Gln_R	GGGGGTGACTGTTCTCCATGAATTCCACC
B998 SETD1B_Glu94Asp_F	AGATCGATGAcTTCTACGTGGGC
B999 SETD1B_Glu94Asp_R	TGAATTTGGGCACCGACA
B1000 SETD1B_113-121_F	tttcacggatgttATGTGCAAGAAGTATGGG
B1001 SETD1B_113-121_R	acttcctgagggacATCATTCAGCTTGGCAAATG
B1004 SETD1B_Thr318Met_F	AGCAAGTTCAtGGACGCCTACAACCGC
B1005 SETD1B_Thr318Met_R	CTCGTGGCGCCGGGCCTT
B1006 SETD1B_Ala1901Glu_F	GGCAACTTCGaGCGCTTCATC
B1007 SETD1B_Ala1901Glu_R	GCACTTGGTGGCGTCGAT
B1008 SETD1B_Ala1901Val_F	GGCAACTTCGtGCGCTTCATC
B1009 SETD1B_Ala1901Val_R	GCACTTGGTGGCGTCGAT
B1010 SETD1B_Glu1948Lys_F	GTTCCCCATCaAGGACGTCAAG
B1011 SETD1B_Glu1948Lys_R	TTATAGTCATAGGTAATCTCCTC
1064_GST_SETD1B_gibson_F	atcggatctggttccgcgtg*gatcc*CTCTCTTCAGCTAAGAAGAAGAAACG
1065_GST_SETD1B_gibson_R	tcgtcagtcagtcacgatgc*ggccgc*CTAGTTGAGGGTCCCCCG
1060_Ash2L_gibson_F	tgacgtcccagactacgcag*ctagc*ATGGCGGCGGCAGGAGCA
1061_Ash2L_gibson_R	tcttggggtcggccctgctg*aattc*TCAGGGTTCCCATGGGGGAC

Supplementary Table S4: Severity scores for all individuals in this study according to their clinical phenotype. Also annotated are functional effects, loss-of-function status, and the domains affected by each variant.

(provided as separate file) <<Supplementary Table S4 Severity scores.xlsx>>

Supplementary References

1. Mencacci, N.E. *et al.* De Novo Mutations in PDE10A Cause Childhood-Onset Chorea with Bilateral Striatal Lesions. *Am J Hum Genet* **98**, 763-71 (2016).
2. Li, H. & Durbin, R. Fast and accurate short read alignment with Burrows-Wheeler transform. *Bioinformatics* **25**, 1754-60 (2009).
3. McKenna, A. *et al.* The Genome Analysis Toolkit: a MapReduce framework for analyzing next-generation DNA sequencing data. *Genome Res* **20**, 1297-303 (2010).
4. Krumm, N. *et al.* Copy number variation detection and genotyping from exome sequence data. *Genome Res* **22**, 1525-32 (2012).
5. Danecek, P. *et al.* The variant call format and VCFtools. *Bioinformatics* **27**, 2156-8 (2011).
6. Chang, C.C. *et al.* Second-generation PLINK: rising to the challenge of larger and richer datasets. *Gigascience* **4**, 7 (2015).
7. Yang, H. & Wang, K. Genomic variant annotation and prioritization with ANNOVAR and wANNOVAR. *Nat Protoc* **10**, 1556-66 (2015).
8. Lek, M. *et al.* Analysis of protein-coding genetic variation in 60,706 humans. *Nature* **536**, 285-91 (2016).
9. Liu, X., Wu, C., Li, C. & Boerwinkle, E. dbNSFP v3.0: A One-Stop Database of Functional Predictions and Annotations for Human Nonsynonymous and Splice-Site SNVs. *Hum Mutat* **37**, 235-41 (2016).
10. Jian, X., Boerwinkle, E. & Liu, X. In silico prediction of splice-altering single nucleotide variants in the human genome. *Nucleic Acids Res* **42**, 13534-44 (2014).
11. Zhang, J. *et al.* BioMart: a data federation framework for large collaborative projects. *Database (Oxford)* **2011**, bar038 (2011).
12. MacDonald, J.R., Ziman, R., Yuen, R.K., Feuk, L. & Scherer, S.W. The Database of Genomic Variants: a curated collection of structural variation in the human genome. *Nucleic Acids Res* **42**, D986-92 (2014).
13. Perenthaler, E. *et al.* Loss of UGP2 in brain leads to a severe epileptic encephalopathy, emphasizing that bi-allelic isoform-specific start-loss mutations of essential genes can cause genetic diseases. *Acta Neuropathol* **139**, 415-442 (2020).
14. Retterer, K. *et al.* Clinical application of whole-exome sequencing across clinical indications. *Genet Med* **18**, 696-704 (2016).
15. Yang, Y. *et al.* Clinical whole-exome sequencing for the diagnosis of mendelian disorders. *N Engl J Med* **369**, 1502-11 (2013).
16. Challis, D. *et al.* An integrative variant analysis suite for whole exome next-generation sequencing data. *BMC Bioinformatics* **13**, 8 (2012).
17. Staufner, C. *et al.* Recurrent acute liver failure due to NBAS deficiency: phenotypic spectrum, disease mechanisms, and therapeutic concepts. *J Inherit Metab Dis* **39**, 3-16 (2016).
18. Shteyer, E. *et al.* Truncating mutation in the nitric oxide synthase 1 gene is associated with infantile achalasia. *Gastroenterology* **148**, 533-536 e4 (2015).
19. Vetro, A. *et al.* Early infantile epileptic-dyskinetic encephalopathy due to biallelic PIGP mutations. *Neurol Genet* **6**, e387 (2020).
20. DePristo, M.A. *et al.* A framework for variation discovery and genotyping using next-generation DNA sequencing data. *Nat Genet* **43**, 491-8 (2011).
21. Liu, X., Jian, X. & Boerwinkle, E. dbNSFP: a lightweight database of human nonsynonymous SNPs and their functional predictions. *Hum Mutat* **32**, 894-9 (2011).
22. Schwarz, J.M., Rodelsperger, C., Schuelke, M. & Seelow, D. MutationTaster evaluates disease-causing potential of sequence alterations. *Nat Methods* **7**, 575-6 (2010).
23. Jagadeesh, K.A. *et al.* M-CAP eliminates a majority of variants of uncertain significance in clinical exomes at high sensitivity. *Nat Genet* **48**, 1581-1586 (2016).

24. Rentzsch, P., Witten, D., Cooper, G.M., Shendure, J. & Kircher, M. CADD: predicting the deleteriousness of variants throughout the human genome. *Nucleic Acids Res* **47**, D886-D894 (2019).
25. Cooper, G.M. *et al.* Distribution and intensity of constraint in mammalian genomic sequence. *Genome Res* **15**, 901-13 (2005).
26. Pollard, K.S., Hubisz, M.J., Rosenbloom, K.R. & Siepel, A. Detection of nonneutral substitution rates on mammalian phylogenies. *Genome Res* **20**, 110-21 (2010).
27. Thorvaldsdottir, H., Robinson, J.T. & Mesirov, J.P. Integrative Genomics Viewer (IGV): high-performance genomics data visualization and exploration. *Brief Bioinform* **14**, 178-92 (2013).
28. Kelly, B.J. *et al.* Churchill: an ultra-fast, deterministic, highly scalable and balanced parallelization strategy for the discovery of human genetic variation in clinical and population-scale genomics. *Genome Biol* **16**, 6 (2015).
29. Koboldt, D.C. *et al.* A de novo nonsense mutation in ASXL3 shared by siblings with Bainbridge-Ropers syndrome. *Cold Spring Harb Mol Case Stud* **4**(2018).
30. Snoeijen-Schouwenaars, F.M. *et al.* Diagnostic exome sequencing in 100 consecutive patients with both epilepsy and intellectual disability. *Epilepsia* **60**, 155-164 (2019).
31. Lee, J.H. & Skalnik, D.G. Rbm15-Mkl1 interacts with the Setd1b histone H3-Lys4 methyltransferase via a SPOC domain that is required for cytokine-independent proliferation. *PLoS One* **7**, e42965 (2012).
32. Alicea-Velazquez, N.L. *et al.* Targeted Disruption of the Interaction between WD-40 Repeat Protein 5 (WDR5) and Mixed Lineage Leukemia (MLL)/SET1 Family Proteins Specifically Inhibits MLL1 and SETd1A Methyltransferase Complexes. *J Biol Chem* **291**, 22357-22372 (2016).
33. Elgert, C., Ruhle, A., Sandner, P. & Behrends, S. Thermal shift assay: Strengths and weaknesses of the method to investigate the ligand-induced thermostabilization of soluble guanylyl cyclase. *J Pharm Biomed Anal* **181**, 113065 (2020).
34. Krzyzewska, I.M. *et al.* A genome-wide DNA methylation signature for SETD1B-related syndrome. *Clin Epigenetics* **11**, 156 (2019).
35. Aref-Eshghi, E. *et al.* Evaluation of DNA Methylation Episignatures for Diagnosis and Phenotype Correlations in 42 Mendelian Neurodevelopmental Disorders. *Am J Hum Genet* **106**, 356-370 (2020).
36. Ritchie, M.E. *et al.* limma powers differential expression analyses for RNA-sequencing and microarray studies. *Nucleic Acids Res* **43**, e47 (2015).
37. Houseman, E.A. *et al.* DNA methylation arrays as surrogate measures of cell mixture distribution. *BMC Bioinformatics* **13**, 86 (2012).
38. Iossifov, I. *et al.* The contribution of de novo coding mutations to autism spectrum disorder. *Nature* **515**, 216-21 (2014).
39. Den, K. *et al.* A novel de novo frameshift variant in SETD1B causes epilepsy. *J Hum Genet* **64**, 821-827 (2019).
40. Hiraide, T. *et al.* De novo variants in SETD1B cause intellectual disability, autism spectrum disorder, and epilepsy with myoclonic absences. *Epilepsia Open* **4**, 476-481 (2019).
41. Hiraide, T. *et al.* De novo variants in SETD1B are associated with intellectual disability, epilepsy and autism. *Hum Genet* **137**, 95-104 (2018).
42. Roston, A. *et al.* SETD1B-associated neurodevelopmental disorder. *J Med Genet* (2020).
